# The revised complete mitogenome sequence of the tree frog *Polypedatesmegacephalus* (Anura, Rhacophoridae) by next-generation sequencing and phylogenetic analysis

**DOI:** 10.7717/peerj.7415

**Published:** 2019-08-01

**Authors:** An Huang, Shuo Liu, Haijun Li, Hongdi Luo, Qingyong Ni, Yongfang Yao, Huailiang Xu, Bo Zeng, Ying Li, Zhimin Wei, Song Li, Mingwang Zhang

**Affiliations:** 1 College of Animal Sciences and Technology, Sichuan Agricultural University, Chengdu, Sichuan Province, China; 2 Farm Animal Genetic Resources Exploration and Innovation Key Laboratory of Sichuan Province, Sichuan Agricultural University, Chengdu, China; 3 Kunming Natural History Museum of Zoology, Kunming Institute of Zoology, Chinese Academy of Sciences, Kunming, Yunnan Province, China; 4 College of Life Science, Sichuan Agricultural University, Yaan, China; 5 Institute of Millet Crops, Hebei Academy of Agriculture and Forestry Sciences, Shijiazhuang, China

**Keywords:** Mitochondrial gene rearrangement, ND5, Phylogenetic relationships, *Polypedates megacephalus*

## Abstract

The mitochondrial genome (mitogenome) sequence of the tree frog *Polypedates megacephalus* (16,473 bp) was previously reported as having the unusual characteristic of lacking the ND5 gene. In this study, a new mitogenome of *P. megacephalus* (19,952 bp) was resequenced using the next-generation sequencing (NGS) and standard Sanger sequencing technologies. It was discovered that the ND5 gene was not lost but translocated to the control region (CR) from its canonical location between the ND4 and ND6 genes. In addition, a duplicated control region was found in the new mitogenome of this species. Conservative region identification of the ND5 gene and phylogenetic analysis confirmed that the ND5 gene was located between two control regions. The phylogenetic relationship among 20 related species of anura revealed a rearrangement of the ND5 gene during the evolutionary process. These results also highlighted the advantages of next-generation sequencing. It will not only decrease the time and cost of sequencing, but also will eliminate the errors in published mitogenome databases.

## Introduction

In general, most vertebrate mitochondrial genomes contain 37 genes, including two ribosomal RNAs (12S and 16S rRNAs), 22 transfer RNAs (tRNAs), and 13 protein-coding genes (PCGs) with a long non-coding control region (CR or D-loop region). The length of this circular structure usually ranges from 15 to 22 kb (Boore, 1999; Han & Zhou, 2005; Kakehashi et al., 2013; Zhang et al., 2018). The gene order of the vertebrate mitogenome is conserved for 37 genes and includes a control region (Boore, 1999; Kurabayashi et al., 2008; Kumazawa et al., 2014). However, studies have increasingly provided experimental and circumstantial evidence for mitogenomic gene rearrangement in invertebrates and vertebrates (Macey et al., 1997; Lavrov, Boore & Brown, 2002; Kumazawa & Endo, 2004; Mabuchi et al., 2004; San Mauro et al., 2006; Xia et al., 2014; Zhang et al., 2018). The variety in gene arrangement suggests that it can be used to estimate the number of divergence events that occur in speciation.

With the rapid development of technology, an increasing number of species’ genomes have been sequenced using the next-generation sequencing (NGS) technology (Lloyd et al., 2012; Yong et al., 2016; Yuan et al., 2016; Jiang et al., 2017). This new method allows us to more accurately explore the details of gene rearrangements in frogs and compare the results with the mitochondrial genome obtained by the conventional sequencing method (Sanger sequencing).

There are approximately 421 species of Rhacophoridae frogs around the world including two subfamilies (Rhacophorinae and Buergeriinae) and 18 genera (Frost, 2018). These frogs are widely distributed in the tropical and subtropical regions of Asia, the southern part of Africa, India, Sri Lanka, Japan, the Philippines, and the Greater Sunda Islands. Although recent studies have illustrated that mitochondrial gene rearrangements have been detected in quite a few species of anura, there is limited data available on the genome of tree frogs and what is available hardly represents the main lineages of Rhacophoridae (Sano et al., 2004; Sano et al., 2005; Huang et al., 2016).

The genus *Polypedates* is widely distributed across Southeast Asia. Due to its morphologically cryptic lineages, the taxonomic status of genus *Polypedates* is disputed (Brown et al., 2010; Blair et al., 2013; Kuraishi et al., 2013; Pan et al., 2013). Brown et al. (2010) sequenced samples of 16S rRNA to investigate the theory that *Polypedates* is actually complex of lineages. Their results showed that the frogs are highly divergent, especially in the mainland areas, but they did not resolve the phylogenetic and taxonomic issues of the complex. Kuraishi et al. (2013) reported that in China *Polypedates* is a monophyletic group that encompasses three distinct taxa. Pan et al. (2013) defined five different lineages in *Polypedates* and four clades, which are distributed in the southern region of China. Most of the studies on this genus focus on phylogenetic analysis of only a few gene fragments (12S rRNA and 16S rRNA). Few scientists have considered the mitochondrial genome in relation to the phylogeny of *Polypedates’s* species.

A previous study has shown that the length of the complete mitogenome of *P. megacephalus* is 16,473 bp with one control region, and it has been reported that the ND5 gene has been lost (Zhang et al., 2005b). Until recently, however, few attempts have been made to verify this unprecedented gene loss. The alleged absence of the ND5 gene has not been reported in any vertebrate mitogenome aside from that of *P*. *megacephalus* (Zhang et al., 2005b) and tuatara (Rest et al., 2003). NADH-ubiquinone oxidoreductase chain 5 is a protein subunit of NADH dehydrogenase located in the mitochondrial inner membrane. The ND5 protein is the largest of the five complexes of the electron transport chain encoded by the ND5 gene (Voet, Voet & Pratt, 2013). A mutant ND5 gene can damage the function of COI and impair any brain or muscle tissue that requires energy input (Charlotte et al., 2010). It is unlikely, therefore, that the mitochondria would lose such an essential gene.

In the present study, we resequenced the complete mitogenome of *Polypedates megacephalus* using Illumina MiSeq sequencing and standard Sanger sequencing. We discovered that the “missing” ND5 gene was not lost but has been translocated to the control region from its canonical location between the tRNA^Glu^ and ND6 genes. Accounting for the ND5 gene and an additional control region, the correct mitogenome of *P. megacephalus* should be 19,952 bp in length instead of 16,473 bp in length. In combination with previously published data from 19 other anuran species, phylogenetic analysis of the newly sequenced mitogenome of *P. megacephalus* suggests that similar ND5 gene rearrangements might have occurred in two distinct ranoid lineages: Dicroglossidae and Rhacophoridae.

## Material and Methods

### Taxon sampling and DNA extraction

The specimen, *P. megacephalus*, was captured in the Shiwandashan National Forest Park, Guangxi Province of China. A field permit (S#2017-42) was granted by the Nature Reserve Administration of Shiwandashan National Forest Park. All animal care and experimental procedures were approved by the Committee of the Ethics on Animal Care and Experiments at Sichuan Agricultural University (CEACE) (Permit Number: S20171001) and carried out in accordance with the guidelines stated by CEACE. Muscle tissue taken from the specimen was preserved in 99% ethanol immediately after collection. Total genomic DNA was extracted from the tissue using an Ezup-type animal genomic DNA extraction kit (Sangon, Shanghai) following the operation manual. The extract was prepared for both Sanger sequencing and next-generation sequencing (NGS).

### Library preparation and mitogenome sequencing

One part of the total DNA sample was sent to Personal Biotechnology (Shanghai, China) for WGS (Whole Genome Shotgun) library construction. Sequencing libraries were generated using the TruSeq DNA Sample Preparation Kit (Illumina, San Diego, CA, USA) and the Template Prep Kit (Pacific Biosciences, Menlo Park, CA, USA). Average insert sizes of approximately 400 bp were prepared with sequencing completed on the Illumina MiSeq sequencing platform producing 251 bp paired-end reads.

**Figure 1:**
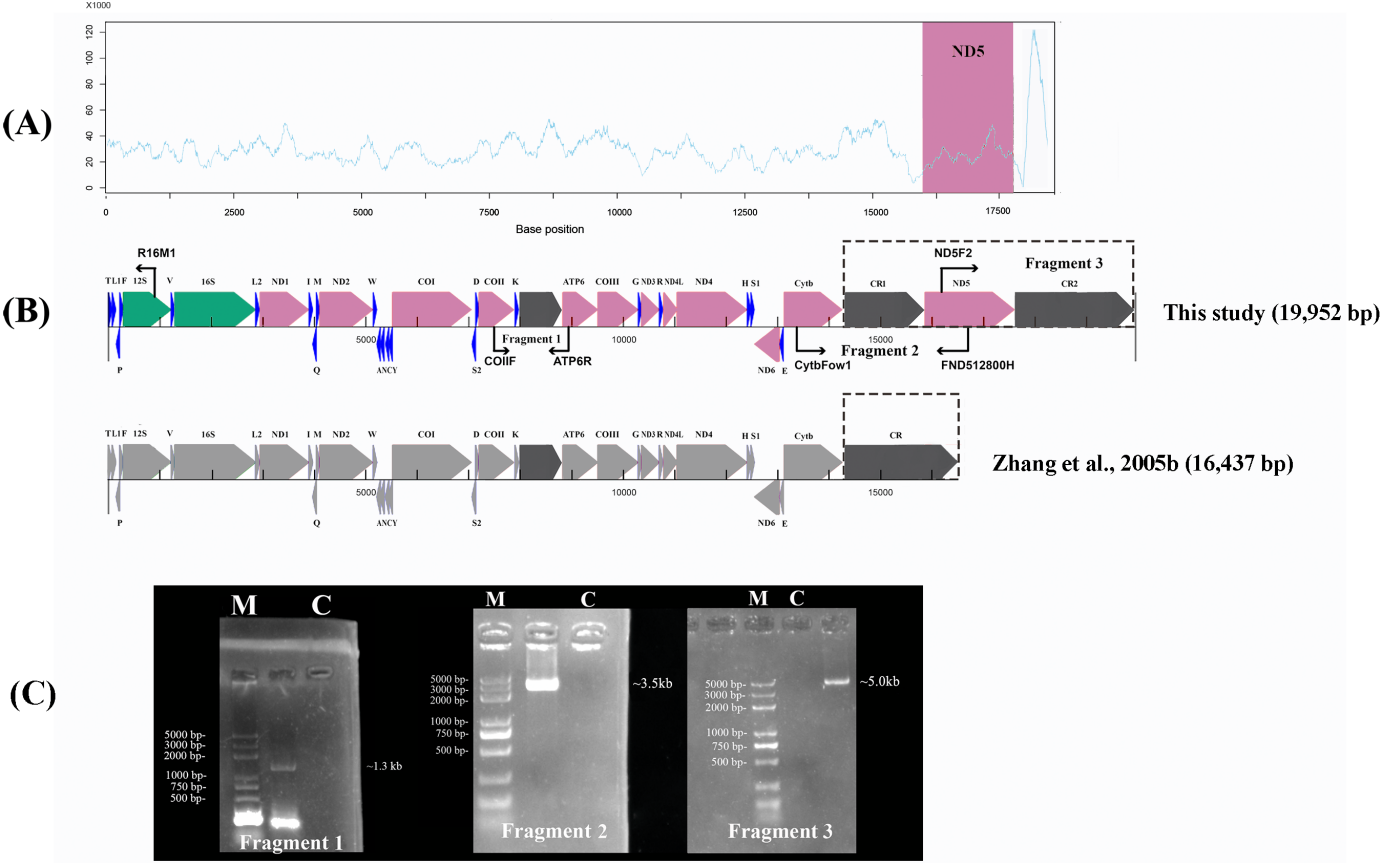
Sanger sequencing and next-generation sequencing coverage of *P. megacephalus* mitogenome. (A) The Illumina reads coverage of the mitogenome represented by sequencing depth. The reads of CR2 were mapped to CR1 as they are similar and caused the NGS results could not align into a complete genome. The red region shows the coverage of ND5 gene is similar to the average coverage on rest of the mitogenome. (B) Schematic of fragment sizes based upon the sequence between COII and ATP6, and the presence of a single mitochondrial control region. The difference between this study and previous study is highlighted by dashed box. (C) A representative agarose gel of LA-PCR products sizes. Single letters C and M represent control and DNA marker, respectively.

To link the gaps where some regions (reads) were not assembled by NGS, the remaining mtDNA was used to perform a long-range PCR (LA-PCR) amplification using two sets of primers: F2 (CytbFow1 and FND512800H) and F3 (ND5F2 and R16M1) (Fig. 1, Table 1). Additionally, in order to verify the authenticity of the non-coding sequence between tRNA^Lys^ and ATP6, we used a set of primers, F1 (COIIF and ATP6R). The primer sets were designed based on the mitogenome sequences obtained from the newly sequenced *P. megacephalus*. The PCR products were preliminarily confirmed on a 1.0% agarose gel (Fig. 1) and cloned using the pEASY-T5 Zero Cloning Kit (TransGen Biotech, Beijing, China). The recombinant plasmids were then sequenced with Sanger sequencing using M13 universal primers. The sequence obtained by Sanger sequencing was then spliced with contigs, or scaffolds, using the ContigExpress software package.

**Table 1:** PCR primer pairs, sequences used in this study.

**Fragment**	**Primer name**	**Nucleotide sequence (5′-3′)**	**Location**
F1	COIIF^a^	GACTCACTCAAGCGTCTATTC	7734
	ATP6R^a^	TGTGGGCGGGTTTATT	9012
F2	CytbFow1^b^	GTYCTMCCNTGRGGHCAAATATCHTTYTG	13510
	FND512800H^c^	CCTATTTTDCGRATRTCYTGYTC	16867
F3	ND5F2^a^	CTCACCCCTCTATTACGACTT	15877
	R16M1^d^	GGGTATCTAATCCCAGTTTG	701

**Notes.**

a Retrieved from this study.

b Sano et al. (2005).

c Zhang et al. (2013).

d Sano et al. (2004).

### Sequence assembly and annotation

The quality of the raw Illumina data (in FASTQ format) was evaluated using FastQC (http://www.bioinformatics.babraham.ac.uk/projects/fastqc/). The raw data was cleaned up, filtered, and assembled. The workflow is described as follows: AdapterRemoval v2 (Schubert, Lindgreen & Orlando, 2016) was used to remove low quality bases and discard the contaminated adapters from the 3′ end of the reads. Then, the short reads were locally corrected by the module SOAPec in SOAP de novo2 (https://sourceforge.net/projects/soapdenovo2/) using the k-mer strategy. We mapped the short reads using BWA software (Li & Durbin, 2009) and SAMtools (Li et al., 2009) (Fig. 1). The sequencing reads have been uploaded to NCBI SRA database (SRA accession number PRJNA516153).

The high quality data was then selected for *de novo* assembly by A5-miseq v20150522 (Coil, Jospin & Darling, 2015) and SPAdes v3.9.0 (Bankevich et al., 2012). The contig of mitogenome sequences was identified by the NT library on NCBI using BLASTn (BLAST C v2.2.31+) and the draft assemblies were corrected by Pilon v1.18 (Walker et al., 2014) to evaluate their accuracy and completeness. The gaps between the ND5 and 12S rRNA by NGS were filled using Sanger sequences of the amplicon generated by the two sets of primers, F2 and F3 (Fig. 1; Table 1).

The assembled mitogenome sequence was annotated using the MITOS web server under the genetic code for vertebrates (http://mitos2.bioinf.uni-leipzig.de/index.py) (Bernt et al., 2013). The secondary structures of tRNAs were also predicted by this web server. The locations of the PCGs and rRNA genes were manually examined by comparisons with the corresponding genes from *Polypedates megacephalus* (AY458598) and *Rhacophorus schlegelii* (AB202078) in MEGA 6.0 (Tamura et al., 2013). The PCG boundaries were identified by ORF Finder (http://www. ncbi.nlm.nih. gov/gorf/gorf.html). The conserved regions of ND5 proteins were identified by Interproscan (http://www.ebi.ac.uk/interpro/) (Mitchell et al., 2018). The mitogenome map was generated by the CGview Server (http://stothard.afns.ualberta.ca/cgview_server/) (Grant & Stothard, 2008). The A+T content and relative synonymous codon usage (RSCU) of the protein-coding genes was calculated by MEGA 6.0 (Tamura et al., 2013). AT skews and GC skews were calculated using the following formulas: AT skew = (A-T)/(A+T), GC skew = (G-C)/(G+C) (Perna & Kocher, 1995).

### Molecular phylogenetic analyses

Phylogenetic analysis was performed using the data of 20 anura mitochondrial genomes, including the one of *P. megacephalus*, generated in this study (Table S1). Another 19 complete mtDNA sequences were retrieved from the GenBank Database, which consisted of seven Dicroglossidae (Liu, Wang & Su, 2005; Ren et al., 2009; Alam et al., 2010; Yu et al., 2012; Li et al., 2014a; Huang & Tu, 2016), four Ranidae (Kakehashi et al., 2013; Li et al., 2014b), three Rhacophoridae (Sano et al., 2004; Sano et al., 2005; Huang et al., 2016), two Hylidae (Zhang et al., 2005a; Igawa et al., 2008), one Mantellinae (Kurabayashi et al., 2008), and two Bombinatoridae (Pabijan et al., 2008) (Table S1); the latter two species (*Bombina bombina* and *B.maxima*) were used as outgroups.

With the exception of the ATP8 gene, the sequences from the 12 concatenated PCGs and 2 rRNAs were chosen for phylogenetic analysis. The analysis was performed using the Bayesian inference (BI) and maximum likelihood (ML) methods. The nucleotide sequences of the 12 PCGs and 2 rRNAs were aligned using MAFFT version 7 (Karen, Marc & John, 2008) and all termination codons were manually deleted. The sequences were edited and trimmed using BioEdit v.7.0.5.3 (Hall, 1999). The best-fit nucleotide substitution models were calculated using PartitionFinder version 2.1.1 (Lanfear et al., 2017) according to the Bayesian information criterion (BIC).

With the appropriate models and partitioning schemes selected in each case (Table S2), a BI phylogenetic tree was constructed using MrBayes v3.2 (Ronquist et al., 2012). In the BI analysis, two independent runs were conducted for 1,000,000 Markov chain Monte Carlo (MCMC) generations with 4 chains (one cold and three heated) and sampling trees occurring every 1,000 generations. The first 25% of trees were discarded as burn-in samples and the remaining trees were used to generate Bayesian consensus trees. For ML analysis, the branch support of ML phylogeny was obtained with 1,000 bootstrap replications in RAxML software under the GTRGAMMA model (Alexandros, 2014), with partitioning parameters similar to those in the BI analysis. The same methods were used for the single gene tree. FigTree 1.4.2 (https://beast.community/figtree) was used to visualize and edit the results of the Bayesian and ML trees.

## Results and Discussion

### Genome composition and gene arrangement

The raw data obtained from the Illumina Pipeline was 524.75 Mb. The total length was 18,328 bp by NGS. However, the NGS results could not be aligned into a complete genome. In order to link the gaps in the NGS sequence, we used the primers Fragment 2 (13,510–16,867) and Fragment 3 (15,877–701) according to the *P. megacephalus* reference mtDNA (GenBank accession no. MH936677) to amplify the remaining sequence segments (Fig. 1; Table 1). LA-PCR amplification and Sanger sequencing with two sets of primers resulted in two products with 3,500-bp and 5,000-bp (Fig. 1; Table 1). Finally, we obtained the complete mitogenome of the *P. megacephalus* with 19,952 bp by merging the sequences with overlapping alignments. The complete mitogenome sequence was deposited in the GenBank databases under the accession number MH936677.

**Figure 2:**
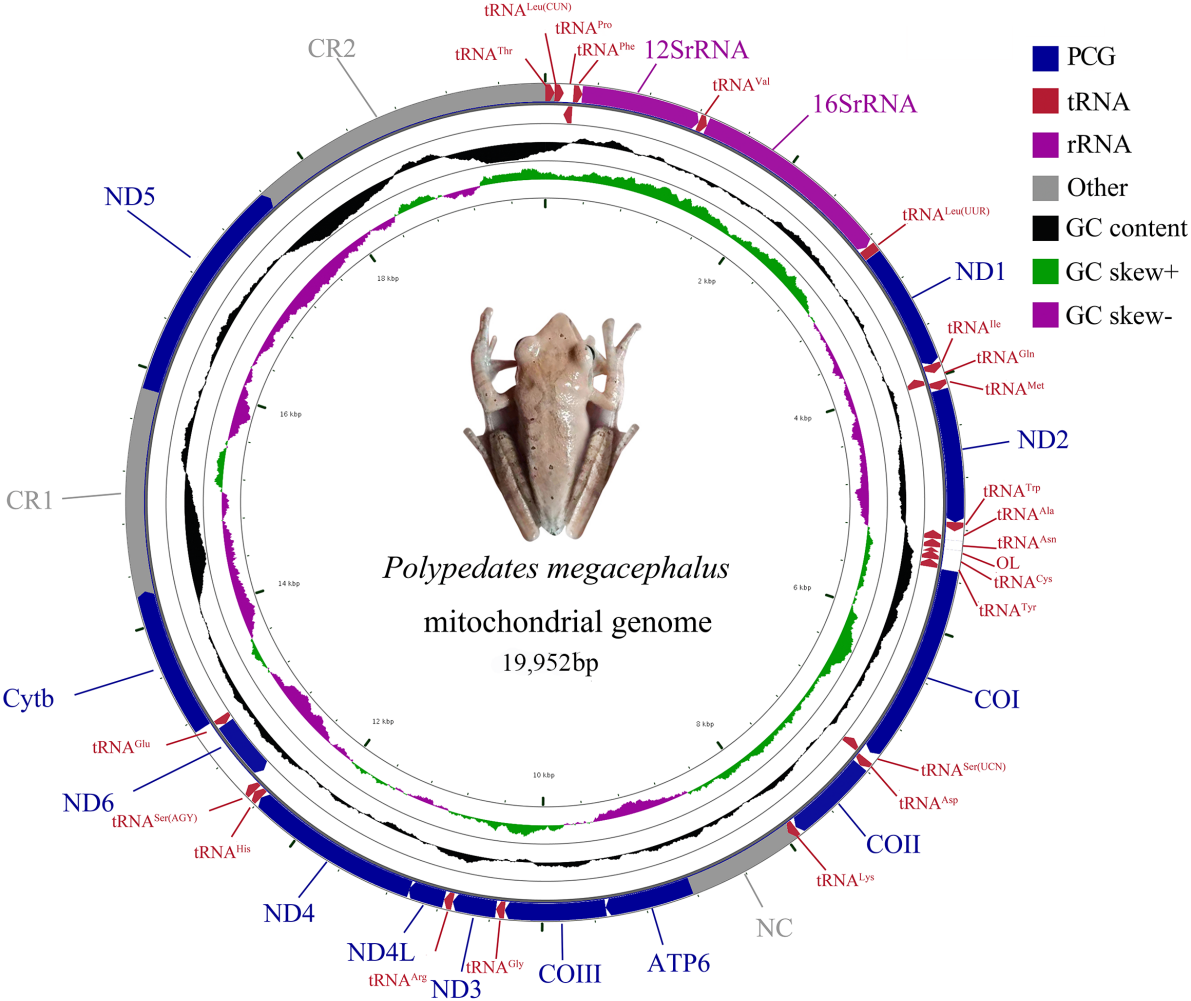
Mitochondrial gene organization of *P. megacephalus*. Genes encoded by the L-strand are on the inner side. O_*L*_ represents the replication original area of L-strand.

The complete mitogenome of *P. megacephalus* was 19,952 bp in total length and contained 22 tRNAs, two rRNAs, 12 PCGs and two control regions (Fig. 2; Table S3). Consistent with other typical amphibian mitogenomes, most of the genes of *P. megacephalus* were coded on the H-strand except for ND6 and 8 tRNAs (Fig. 2; Table S3). 22 tRNAs with sizes ranging from 65 to 74 bp were interspersed across the mitogenome. Aside from tRNA^Cys^ and tRNA^Ser(AGY )^, which cannot possess a perfect cloverleaf structure (Fig. S1), these tRNAs (20 of 22) can fold into a complete secondary structure. In addition, there was a notable noncoding region that was 862 bp in length between tRNA^Lys^ and ATP6 genes in the mitogenome. This was consistent with the results from a previous study (Zhang et al., 2005b) (Table S3). The base compositions of the whole mitochondrial genome, 2 rRNA genes, PCGs, and control regions of *P. megacephalus* are shown in Table S4. The A + T content for the PCGs ranged from 54.4% (ND3) to 63.1% (ND2). Four PCGs (ND1, ND2, ATP6, ND4,) had an A+T content of over 60%. The A +T content of the CR1 and CR2 were 65.7% and 68.8%, respectively. The AT-skew value was negative for the entire mitogenome as well as for most of the genes with the exception of positive values for 5 genes (12S rRNA, 16S rRNA, ND2, COII, ND5, ND6). The GC-skew values for the whole genome, PCGs, 2 rRNA genes, and control regions were negative (0.113 to 0.524), indicating a bias toward the use of Cs over Gs.

The gene arrangement of the *P. megacephalus* mitogenome diverged from that of typical vertebrates (Fig. 2; Table S3). Impressively, the ND5 gene (1,779 bp) was detected and subsequently located between two control regions (CRs) after the gene had been thought to be lost in this species (Zhang et al., 2005b). The ND5 gene and two CRs (CR1 and CR2) accompanied by the tRNA gene cluster of tRNA^Leu(CUN)^/tRNA^Thr^/tRNA^Pro^/tRNA^Phe^ (LTPF cluster) were also identified in the Schlegel’s tree frog, *Rhacophorus schlegelii* (Sano et al., 2005).

### Protein-coding genes

The total length of all 12 PCGs was 11,121 bp and the overall A+T content was 59.2%. Most genes were encoded by the H-strand except for ND6. The initiation codons of most of the PCGs were ATG, except ND2 began with ATT, ND4 with GTG, and COI, COII, and ATP6 with ATA. Two genes (COI and ND6) ended with AGG as the stop codon, three genes (COII, ND4L and ND5) used the TAA codon, and the ND2 gene terminated with the TAG codon. The remaining six PCGs (ND1, ATP6, COIII, ND3, ND4 and Cytb) ended with an incomplete stop codon, T–. This incomplete codon can be transformed into a complete one (TAA) through transcription (Ojala et al., 1981).

The relative synonymous codon usage (RSCU) values are shown in Fig. S2. Compared to the other synonymous codons, the RSCU analysis indicated that codons including A or T at the third position were always overused. The most frequently encoded amino acids were Leu (CUN), Ile, and Ala (>300), while the least frequently used amino acid was Cys (<45).

**Figure 3:**
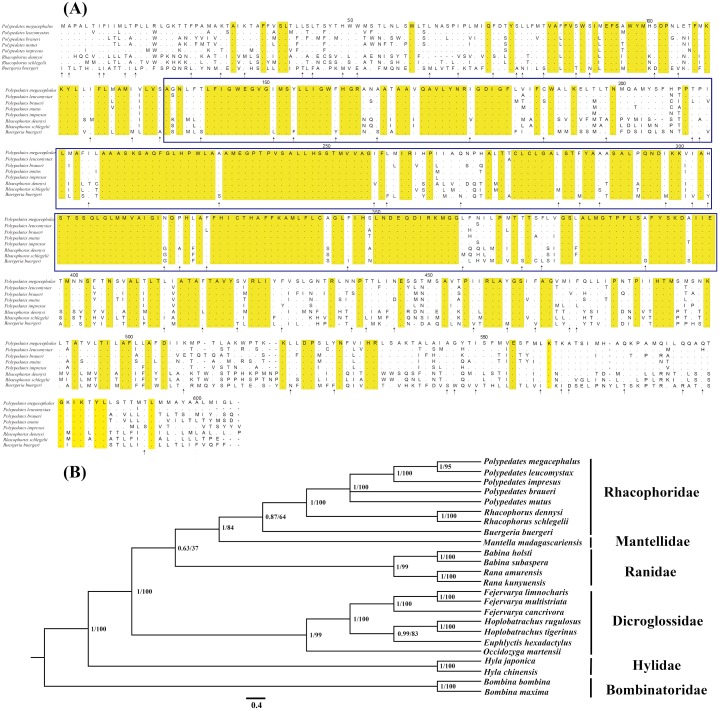
Evidences of the existence of ND5 gene in the mitogenome of *P. megacephalus*. (A) Comparison of amino acids of the ND5 with the corresponding amino acid sequences of seven other Rhacophoridae species. The highlighted section in yellow indicates an amino acid conserved in all eight sequences; an arrow indicates amino acids conserved in 7/8 sequences, and dashes indicate indels. The functionally conserved regions of ND5 proteins are boxed. (B) Phylogenetic results of BI and ML analysis among 24 related frogs using single ND5 gene. NCBI accession number: *P. leucomystax* (MK622898), *P. impresus* (MK622901), *P. braueri* (MK6 87567), *P. mutus* (MK622900).

In this study, we discovered that the previously reported “absent” ND5 gene was not actually absent but was instead translocated to the position between the CR1 and CR2. To further confirm our results, the ND5 gene of four other *Polypedates* frogs was also obtained using Sanger sequencing (Dataset S3). The translocated ND5 is an intact gene that has both a normal initiation and stop codon and can be translated into proteins. The open reading frame was also identified by ORF Finder on the NCBI website and the membrane transport proteins of NADH-ubquinone oxidoreductase 5 were identified by Interproscan. Among the aligned sequences of the ND5 gene from eight tree frogs, the functionally conserved region was the most prominent with respect to amino acid sequence similarity (Fig. 3A). The phylogenetic results of the ND5 gene from eight tree frogs also revealed that the closely related species were clustered into the correct clades (Fig. 3B).

Gene rearrangement is generally attributed to a slipped-strand mispairing caused by the stem loop structure of mitochondria that leads to gene duplication. Due to natural selection and the accumulation of natural mutations, the repeat gene sequences were excised in subsequent random loss (Macey et al., 1997). The hypothetical gene rearrangement processes of the tRNAs and ND5 as proposed by Sano (Sano et al., 2005) are shown in Fig. S3: the first tandem duplication occurs in the ND5-CR. After the repeat genes are deleted, the ND5 gene is transferred downstream of the CR. The secondary tandem duplication occurs in the CR-ND5; two CRs appear after the deletion of the repeat ND5 gene. The third tandem duplication occurs in the LTPF cluster (tRNA^Leu(CUN)^/tRNA^Thr^/ tRNA^Pro^/ tRNA^Phe^).

In some species of Dicroglossidae and Rhacophoridae, the position of the ND5 gene shifts to a different locality. For instance, (1) in *Fejervarya limnocharis* (Liu, Wang & Su, 2005) and *F. cancrivora* (Ren et al., 2009), the ND5 gene shifts to the 3′end of the CR. Ren et al. (2009) deduced that after a duplication of the ND5-CR region, the ND5 gene, which was located upstream of the CR, was deleted and the downstream ND5 gene is preserved as the CR-ND5 arrangement in the genus *Fejervarya.* (2) The ND5 gene of *Rhacophorus schlegelii* (Sano et al., 2005) is translocated between two copied CRs, confirming the evolutionary trend of this unique gene order. Therefore, we infer that the ND5 gene of *P. megacephalus* might have experienced a similar gene rearrangement event.

The ATP8 gene is a gene that encodes a subunit of mitochondrial ATP synthase (Doering, Ermentrout & Oster, 1995). The published gene sequences of the *P. megacephalus* (Zhang et al., 2005b) and *P. braueri* (Liu et al., 2015) reflected a loss of the ATP8 gene in the *Polypedates* species. Our results also detected no ATP8 gene or ATP8-like sequence in *P. megacephalus*.

The ATP8 gene has a high mutation rate, but still possesses the MPQL amino acids as the conserved motif at the N-terminus of the typical metazoan ATP8 (Gissi, Iannelli & Pesole, 2008; Ulianosilva et al., 2015). However, the putative sequence of *P. megacephalus* was unable to be translated into its corresponding protein suggesting this gene might have lost its function. The absence of ATP8 has been detected in several phylogenetically distant metazoan species: Nematoda (Lavrov & Brown, 2001), Mollusca (Gissi, Iannelli & Pesole, 2008; Ulianosilva et al., 2015), and Rotifera (Steinauer et al., 2005; Suga et al., 2008). The majority of these are invertebrates. Among the vertebrates, however, the absence was only found in the *Polypedates* species (Zhang et al., 2005b; Liu et al., 2015)*.* To verify the authenticity of the lost ATP8 (in case of sequencing errors), we also sequenced this region in four other species of the genus *Polypedates*, finding that there was indeed a noncoding sequence between the tRNA^Lys^ and ATP6 genes (Dataset S3). It is likely that the ATP8 gene has become a pseudogene as Zhang et al. (2005b) inferred (Fig. S4).

### Noncoding regions

The noncoding regions in *P. megacephalus* mtDNA included the control regions and a few intergenic spacers.

**Figure 4:**
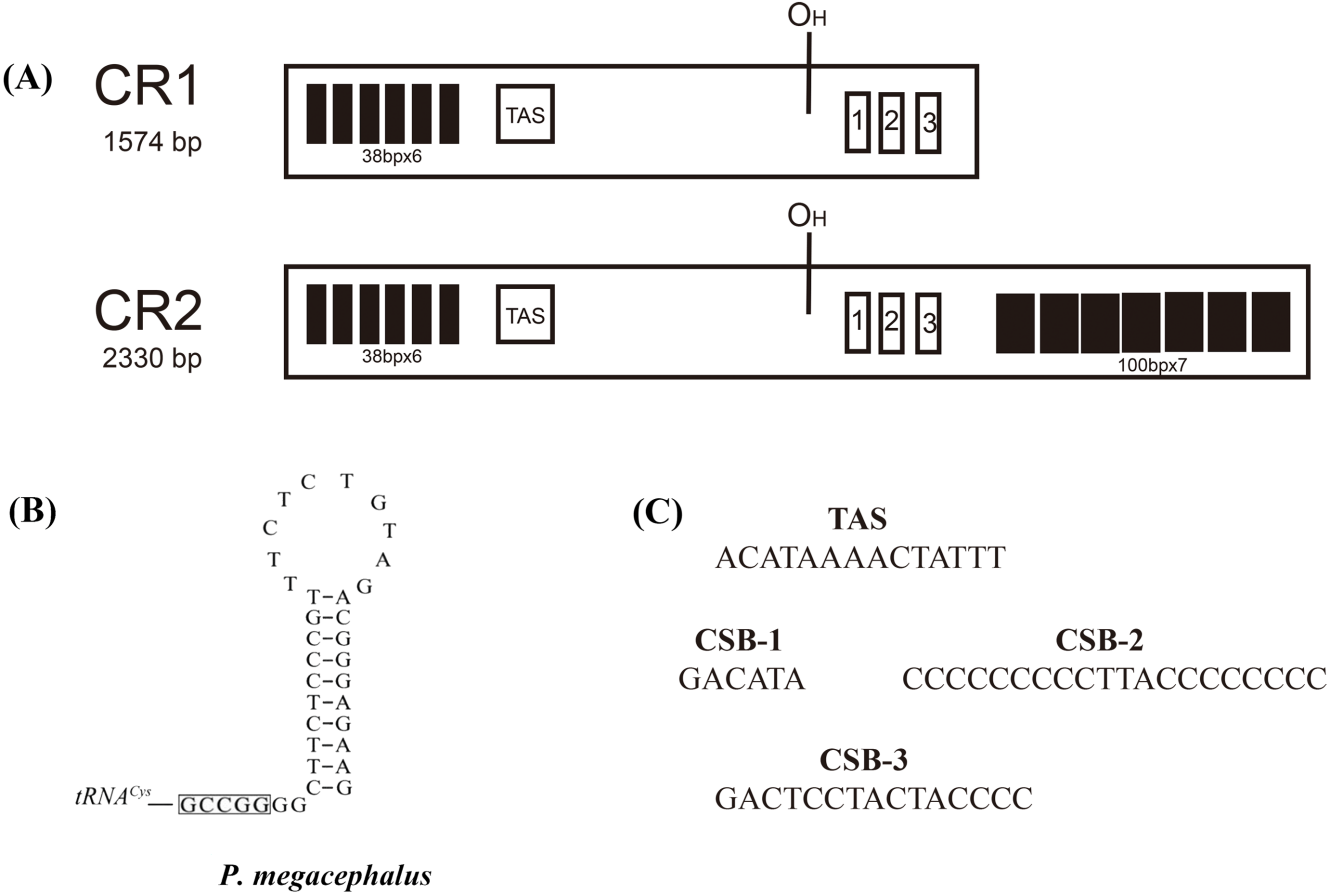
Structures of *P. megacephalus* control regions. (A) These regions consist of tandem repeat units, termination-associated sequences (TAS), H-stand origin of replication (O_*H*_) and conserve sequence block 1, 2, 3 (CSB1, CSB2, CSB3). (B) The putative secondary structures of the replication original area of L-strand. The 5′-GCCGG-3′ sequence motif as the base of the stem within the tRNA^*Cys*^. (C) Nucleotide sequences of conserved elements.

Two major noncoding regions (CR1 and CR2) were found in the *P. megacephalus* mitogenome (Fig. 2; Table S3). CR1, which had a total length of 1,574 bp, was located between the Cytb and ND5 genes in a position homologous to that of the CR in the *Buergeria buergeri* mitogenome (Sano et al., 2004) and CR1 in the *Rhacophorus schlegelii* (Sano et al., 2005). CR2, which was 2,330 bp, was located between the ND5 and tRNA^Thr^ genes (Fig. 2). Two CRs were separated by the ND5 gene with a nucleotide sequence similarity of 99% over 1,571bp. Six tandem repeat units of 38 bp were detected in the 5′ side of CR1 and CR2, but the 3′ side of CR2 had more 760 bp repeat sequences than CR1 (Fig. 4). These regions are characterized by lower conservation, a high degree of variation, transcription regulation, and the replication of mtDNA. Duplicated control regions have been reported in several metazoan lineages in species such as the tree frog *Rhacophorus schlegelii* (Sano et al., 2005), several major snake families (Kumazawa et al., 1998; Dong & Kumazawa, 2005), Reptile tuatara (Rest et al., 2003), *Thalassarche* albatrosses (Abbott et al., 2005), Australasian ixodes ticks (Shao et al., 2005), and Antarctic notothenioids (Zhuang & Cheng, 2010). Two CR copies can improve the transcription and translation efficiency of mitochondrial encoded respiratory chain proteins (Zhuang & Cheng, 2010). Some studies report that the two CR sequences are quite similar, suggesting that they may be undergoing concerted evolution (Kumazawa et al., 1998; Forcada et al., 2003; Peng, Nie & Pu, 2006).

However, consistent with Zhang’s experimental results (Zhang et al., 2005b), an 862 bp noncoding sequence (NC) was observed between the tRNA^Lys^ and ATP6 genes. This NC might have replaced the original position of the ATP8 gene (Fig. 2 and Fig. S4).

### Phylogenetic analyses

**Figure 5:**
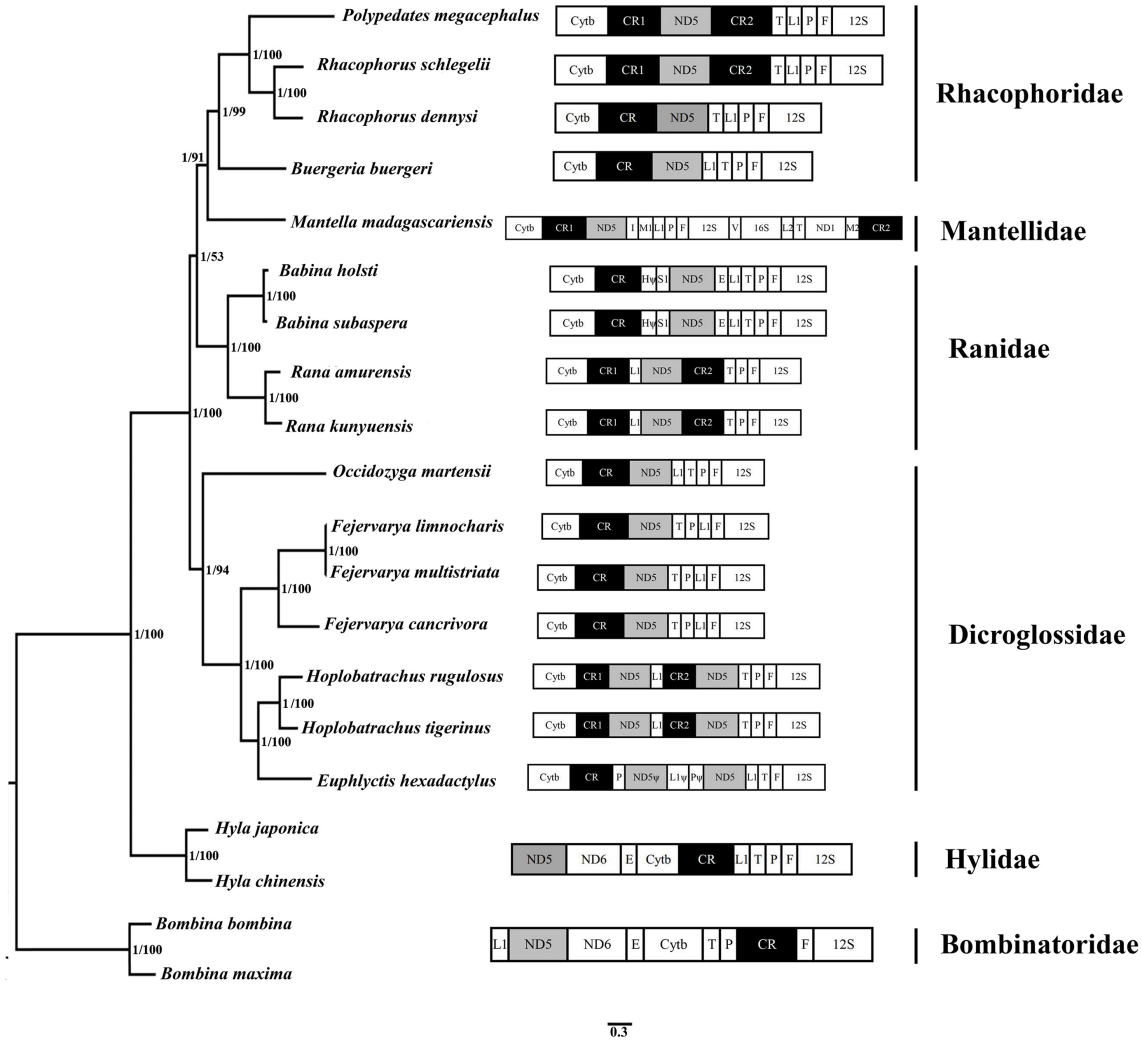
Phylogenetic tree constructed based on the nucleotide dataset of 12 PCGs and 2 rRNAs using Bayesian inference and ML method. The PCGs are shown in abbreviations. tRNA genes are represented by the standard single amino acid code, the rearranged ND5 and CR are in shed boxes.

The BI and ML methods of phylogenetic reconstruction yielded fully congruent tree topologies which supported the previous classification (Fig. 5) (Frost et al., 2006; Kakehashi et al., 2013; Kurabayashi & Sumida, 2013; Zhang et al., 2013; Li et al., 2014a; Xia et al., 2014; Yuan et al., 2016).

20 species of anura in the phylogenetic trees were clustered into 6 branches. In this study, phylogenetic trees based on 12 PCGs and 2 rRNAs strongly supported monophyly of Rhacophoridae and Dicroglossidae (BPP = 1 and bootstrap value = 100). Additionally, the relationships among the three ranid families (Mantellidae, Rhacophoridae, and Ranidae) were well resolved in BI analysis (BPP = 1). Some previous studies have suggested the family Ranidae is a paraphyletic group with respect to Mantellidae and Rhacophoridae (Igawa et al., 2008; Kurabayashi et al., 2008; Ren et al., 2009) and supported Dicroglossidae as being in a sister clade relationship with (Ranidae, (Mantellidae, Rhacophoridae)) (Zhang et al., 2013; Li et al., 2014a; Chen et al., 2017).

The tRNA^Leu(CUN)^/tRNA^Thr^/ tRNA^Pro^/ tRNA^Phe^ (LTPF cluster) was arranged like the typical gene sequence of *Babina* in Ranidae, but in two *Rana* species (*Rana amurensis* and *R. kunyuensis*), the tRNA^Leu(CUN)^ moved downstream of one of the CRs (Fig. 5). The ND5 genes of the species of these two genera had both moved. Kurabayashi et al. (2008) sequenced the complete mitogenome of *Mantella madagascariensis* and partial fragments of the Cytb–ND2 region in other Mantellidae-related frogs, and also determined that the ND5 gene had shifted downstream of the CR. The LTPF clusters of some species of Rhacophoridae are arranged in the order of TLPF (tRNA^Thr^/ tRNA^Leu(CUN)^/ tRNA^Pro^/ tRNA^Phe^); moreover, the ND5 gene is again located downstream of the CR (Fig. 5).

Four tRNA genes were arranged as TPLF (tRNA^Thr^/tRNA^Pro^/ tRNA^Leu(CUN)^/ tRNA^Phe^) in some species of Dicroglossidae (*F. cancrivora*, *F. limnocharis* and *F. multistriata*), and here the ND5 gene was also translocated downstream of the CR (Fig. 5). Notably, the duplicated ND5 gene was detected in two species (*H. rugulosus and H. tigerinus*), further confirming the hypothesis of mitochondrial tandem duplication. Therefore it seems that the translocation of the ND5 gene took place in the common lineage of these Dicroglossidae genera ancestors (Alam et al., 2010; Chen et al., 2017). Based on the phylogenetic results it appears that the same translocation of the ND5 gene occurred in the common ancestor of Mantellidae as well as Rhacophoridae, as well as the common ancestral lineage of four genera of Dicroglossidae (*Occidozyga*, *Fejervarya*, *Hoplobatrachus* and *Euphlyctis*) (Chen et al., 2017). As discussed above, the same gene rearrangements probably occurred in two distinct ranoid lineages as convergent genetic evolutionary events (Ren et al., 2009; Chen et al., 2017).

### Possible causes for misdiagnosis of an “absent” ND5 gene

It was previously reported that the ND5 gene was absent in the mitogenome of *P. megacephalus* (16,473 bp) (Zhang et al., 2005b), which conflicts with the present study. The presence of the ND5 gene was observed in the mitogenome of the same species (19,952 bp) with an extra CR. In reanalyzing Zhang’s data, three factors could explain the why Zhang’s results contradict the present study. (1) Two primer sets (LX16S1/LX11932H [∼7 kb] and LX9844/LX16S1R [∼9 kb]) for their long-range PCR amplifications were designed to amplify the entire mitogenome. Each sequence of the two long PCR fragments overlapped by about 3 kb. However, location verification of the primer pair (LX9844/LX16S1R [∼9 kb]) showed that the fragment size should be 14 kb rather than 9 kb. (2) It was previously inferred that the ND5 gene was absent based on the amplified sequence of the mtDNA region from tRNA^Lys^ to Cytb from *P. megacephalus* and other related frogs (Zhang et al., 2005b). It was also reported that the amplified fragments from these tree frogs were apparently shorter than those of other frogs, suggesting the absence of the ND5 gene from its original position. However, this study indicates that this evidence could be attributed to translocation instead of gene loss. (3) In addition, it was found that the copied CRs in *P. megacephalus*’s mitogenome exhibited highly similar sequences to one another at the 5′ region (100% across 38 × 6 = 228 bp) (Fig. 4 and Fig. S5), which may have resulted in assembly error (i.e., the 5′ region of CR1 and CR2 were misassembled or combined as a single CR). Thus, only one CR was observed and the absence of the ND5 gene was reported (Zhang et al., 2005b). The misdiagnosis of an “absent” ND5 gene could be attributed to the errors in the estimation of long-range PCR fragment size, assembly, and alignment.

## Conclusion

We successfully resequenced and revised the entire mitochondrial genome sequence of *Polypedates megacephalus* and found the ND5 gene located between two control regions, although a previous study had suggested this gene to be absent. Our experiments indicated that more accurate results can be obtained using Sanger sequencing in combination with next-generation sequencing. Our subsequent experiments suggest that all species of genus *Polypedates* might have the same mitochondrial gene arrangement. Further investigations focusing on the mitochondrial gene arrangement of other tree frogs will enhance the understanding of the molecular mechanisms and evolutionary history behind the phylogenetic pathway.

##  Supplemental Information

10.7717/peerj.7415/supp-1 Dataset S1 Raw data for the complete mitogenome sequence of *Polypedates megacephalus* in fasta format

10.7717/peerj.7415/supp-2 Dataset S2 The complete mitochondrial sequence of *Polypedates megacephalus*

10.7717/peerj.7415/supp-3 Dataset S3 Partial sequences of five species of polypedates frogs Evidence of the existence of ND5 and non-coding sequences.

10.7717/peerj.7415/supp-4 Figure S1 Putative secondary structures of tRNA genes of *P. megacephalus* mitochondrial genome

10.7717/peerj.7415/supp-5 Figure S2 Relative synonymous codon usage (RSCU) in *P. megacephalus* mitochondrial genome Codon families are provided on the *x-axis* while RSCU are shown on the *y-axis*.

10.7717/peerj.7415/supp-6 Figure S3 The gene rearrangement processes of tRNAs and the hypothetical mechanism of transfered ND5 based on the inference of Sano et al. (2005) The thick solid lines represent replicated genes, dashed lines denote deleted genes, respectively.

10.7717/peerj.7415/supp-7 Figure S4 Presumptive mechanism of the forming of the noncoding regions in *P. megacephalus* based on previous hypothesis (*Zhang et al., 2005*) Sequence alignment results shows the noncoding region have high similarity with tRNA_*Lys*_ which probably became a pseudogene.

10.7717/peerj.7415/supp-8 Figure S5 Comparative alignment of the control regions of the two *P. megacephalus* individuals Previous sequence was marked by an asterisk (*). Dots represent corresponding nucleotides are identical while dashes indicate gaps in the sequence. Several components of CR are boxed. The downstream of CR* has high similarity with downstream of CR2.

10.7717/peerj.7415/supp-9 Table S1 Mitochondrial genome information of Anura species from GenBank

10.7717/peerj.7415/supp-10 Table S2 The best models of partitioning schemes selected by PartitionFinder for BI analysis

10.7717/peerj.7415/supp-11 Table S3 Location of features in the mtDNA of *P. megacephalus*

10.7717/peerj.7415/supp-12 Table S4 Base compositions of the mitochondrial genome of *Polypedates megacephalus*

## Additional Information and Declarations

### Field Study Permissions

The following information was supplied relating to field study approvals (i.e., approving body and any reference numbers): Field permit S#2017-42 was granted by the Nature Reserve Administration of Shiwandashan National Forest Park.

### Data Availability

The following information was supplied regarding the deposition of DNA sequences: The mitochondrial sequence of Polypedates megacephalus described here are accessible via GenBank accession number MH936677 and the Sequence Read Archive (SRA) is PRJNA516153. Raw data of the complete mitogenome sequence of tree frog Polypedates megacephalus is available at Figshare: Huang, An (2019): Polypedates megacephalus_PE400.fastq.gz. figshare. Dataset. https://doi.org/10.6084/m9.figshare.7935194.v1.

## References

[ref-1] Abbott CL, Double MC, Trueman JW, Robinson A, Cockburn A (2005). An unusual source of apparent mitochondrial heteroplasmy: duplicate mitochondrial control regions in Thalassarche albatrosses. Molecular Ecology.

[ref-2] Alam MS, Kurabayashi A, Hayashi Y, Sano N, Khan MMR, Fujii T, Sumida M (2010). Complete mitochondrial genomes and novel gene rearrangements in two dicroglossid frogs, Hoplobatrachus tigerinus and Euphlyctis hexadactylus, from Bangladesh. Genes & Genetic Systems.

[ref-3] Alexandros S (2014). RAxML version 8: a tool for phylogenetic analysis and post-analysis of large phylogenies. Bioinformatics.

[ref-4] Bankevich A, Nurk S, Antipov D, Gurevich AA, Dvorkin M, Kulikov AS, Lesin VM, Nikolenko SI, Pham S, Prjibelski AD (2012). SPAdes: a new genome assembly algorithm and its applications to single-cell sequencing. Journal of Computational Biology.

[ref-5] Bernt M, Donath A, Jühling F, Externbrink F, Florentz C, Fritzsch G, Pütz J, Middendorf M, Stadler PF (2013). MITOS: improved de novo metazoan mitochondrial genome annotation. Molecular Phylogenetics & Evolution.

[ref-6] Blair C, Davy CM, Ngo A, Orlov NL, Shi HT, Lu SQ, Gao L, Rao DQ, Murphy RW (2013). Genealogy and demographic history of a widespread amphibian throughout indochina. Journal of Heredity.

[ref-7] Boore JL (1999). Animal mitochondrial genomes. Nucleic Acids Research.

[ref-8] Brown RM, Linkem CW, Siler CD, Sukumaran J, Esselstyn JA, Diesmos AC, Iskandar DT, Bickford D, Evans BJ, McGuire JA, Grismer L, Supriatna J, Andayani N (2010). Phylogeography and historical demography of Polypedates leucomystax in the islands of Indonesia and the Philippines: evidence for recent human-mediated range expansion?. Molecular Phylogenetics and Evolution.

[ref-9] Charlotte LA, Monika M, Christopher R, Iain PH, Simon ASP, John ML, Simon JH, Rita H, Helen M, Robert WT (2010). A novel mitochondrial MTND5 frameshift mutation causing isolated complex I deficiency, renal failure and myopathy. Neuromuscular Disorders: NMD.

[ref-10] Chen Z, Li H, Zhu Y, Feng Q, He Y, Chen X (2017). Molecular phylogeny of the family Dicroglossidae (Amphibia: Anura) inferred from complete mitochondrial genomes. Biochemical Systematics and Ecology.

[ref-11] Coil D, Jospin G, Darling AE (2015). A5-miseq: an updated pipeline to assemble microbial genomes from Illumina MiSeq data. Bioinformatics.

[ref-12] Doering C, Ermentrout B, Oster G (1995). Rotary DNA motors. Biophysical Journal.

[ref-13] Dong S, Kumazawa Y (2005). Complete mitochondrial DNA sequences of six snakes: phylogenetic relationships and molecular evolution of genomic features. Journal of Molecular Evolution.

[ref-14] Forcada I, Mur M, Mora E, Vieta E, Bartrés-Faz D, Portella MJ (2003). Evolution of the deep-sea gulper Eel mitochondrial genomes: large-scale gene rearrangements originated within the Eels. Molecular Biology and Evolution.

[ref-15] Frost DR (2018). http://research.amnh.org/herpetology/amphibia/index.html.

[ref-16] Frost DR, Grant T, Faivovich J, Bain RH, Haas A, Haddad C, RO de SÁ, Channing A, Wilkinson M, Donnellan SC (2006). The amphibian tree of life. Bullammusnatlhist.

[ref-17] Gissi C, Iannelli F, Pesole G (2008). Evolution of the mitochondrial genome of Metazoa as exemplified by comparison of congeneric species. Heredity.

[ref-18] Grant JR, Stothard P (2008). The CGView server: a comparative genomics tool for circular genomes. Nucleic Acids Research.

[ref-19] Hall TA (1999). BioEdit: a user-friendly biological sequence alignment editor and analysis program for Windows 95/98/NT. Nucleic Acids Symposium Series.

[ref-20] Han DM, Zhou KY (2005). Complete sequence and gene organization of the mitochondrial genome of Tokay (Gekko gecko). Zoological Research.

[ref-21] Huang M, Tong L, Duan R, Zhang S, Li H (2016). The complete mitochondrial genome of Rhacophorus dennysi (Anura: Rhacophoridae) and phylogenetic analysis. Mitochondrial DNA Part A DNA Mapping Sequencing & Analysis.

[ref-22] Huang ZH, Tu FY (2016). Mitogenome of Fejervarya multistriata: a novel gene arrangement and its evolutionary implications. Genetics and Molecular Research.

[ref-23] Igawa T, Kurabayashi A, Usuki C, Fujii T, Sumida M (2008). Complete mitochondrial genomes of three neobatrachian anurans: a case study of divergence time estimation using different data and calibration settings. Gene.

[ref-24] Jiang L, Zhao L, Cheng D, Zhu L, Zhang M, Ruan Q, Chen W (2017). The complete mitochondrial genome sequence of the Sichuan Digging Frog, Kaloula rugifera (Anura: Microhylidae) and its phylogenetic implications. Gene.

[ref-25] Kakehashi R, Kurabayashi A, Oumi S, Katsuren S, Hoso M, Sumida M (2013). Mitochondrial genomes of Japanese Babina frogs (Ranidae, Anura): unique gene arrangements and the phylogenetic position of genus Babina. Genes & Genetic Systems.

[ref-26] Karen MW, Marc AS, John PH (2008). Alignment uncertainty and genomic analysis. Science.

[ref-27] Kumazawa Y, Endo H (2004). Mitochondrial genome of the Komodo dragon: efficient sequencing method with reptile-oriented primers and novel gene rearrangements. DNA Research.

[ref-28] Kumazawa Y, Miura S, Yamada C, Hashiguchi Y (2014). Gene rearrangements in gekkonid mitochondrial genomes with shuffling, loss, and reassignment of tRNA genes. BMC Genomics.

[ref-29] Kumazawa Y, Ota H, Nishida M, Ozawa T (1998). The complete nucleotide sequence of a snake (Dinodon semicarinatus) mitochondrial genome with two identical control regions. Genetics.

[ref-30] Kurabayashi A, Sumida M (2013). Afrobatrachian mitochondrial genomes: genome reorganization, gene rearrangement mechanisms, and evolutionary trends of duplicated and rearranged genes. BMC Genomics.

[ref-31] Kurabayashi A, Sumida M, Yonekawa H, Glaw F, Vences M, Hasegawa M (2008). Phylogeny, recombination, and mechanisms of stepwise mitochondrial genome reorganization in mantellid frogs from Madagascar. Molecular Biology and Evolution.

[ref-32] Kuraishi N, Matsui M, Hamidy A, Belabut DM, Ahmad N, Panha S, Sudin A, Yong HS, Jiang J-P, Ota H, Thong HT, Nishikawa K (2013). Phylogenetic and taxonomic relationships of the Polypedates leucomystaxcomplex (Amphibia). Zoologica Scripta.

[ref-33] Lanfear R, Frandsen PB, Wright AM, Senfeld T, Calcott B (2017). PartitionFinder 2: new methods for selecting partitioned models of evolution for molecular and morphological phylogenetic analyses. Molecular Biology & Evolution.

[ref-34] Lavrov DV, Boore JL, Brown WM (2002). Complete mtDNA sequences of two millipedes suggest a new model for mitochondrial gene rearrangements: duplication and nonrandom loss. Molecular Biology & Evolution.

[ref-35] Lavrov DV, Brown WM (2001). Trichinella spiralis mtDNA: a nematode mitochondrial genome that encodes a putative ATP8 and normally structured tRNAS and has a gene arrangement relatable to those of coelomate metazoans. Genetics.

[ref-36] Li H, Durbin R (2009). Fast and accurate short read alignment with Burrows-Wheeler transform. Bioinformatics.

[ref-37] Li H, Handsaker B, Wysoker A, Fennell T, Ruan J, Homer N, Marth G, Abecasis G, Durbin R, Genome project data processings (2009). The sequence alignment/map format and SAMtools. Bioinformatics.

[ref-38] Li E, Li X, Wu X, Feng G, Zhang M, Shi H, Wang L, Jiang J (2014a). Complete nucleotide sequence and gene rearrangement of the mitochondrial genome of Occidozyga martensii. Journal of Genetics.

[ref-39] Li J, Yin W, Xia R, Lei G, Fu C (2014b). Complete mitochondrial genome of a brown frog, (Anura: Ranidae). Mitochondrial DNA.

[ref-40] Liu ZQ, Wang YQ, Su B (2005). The mitochondrial genome organization of the rice frog, Fejervarya limnocharis (Amphibia: Anura): a new gene order in the vertebrate mtDNA. Gene.

[ref-41] Liu Y, Yang M, Jiang Y, Han F, Li Y, Ni Q, Yao Y, Xu H, Zhang M (2015). The near complete mitochondrial genome of white-lipped Treefrog, Polypedates braueri (Anura, Rhacophoridae). DNA Sequence.

[ref-42] Lloyd RE, Foster PG, Matthew G, Littlewood DTJ (2012). Next generation sequencing and comparative analyses of Xenopus mitogenomes. BMC Genomics.

[ref-43] Mabuchi K, Miya M, Satoh TP, Westneat MW, Nishida M (2004). Gene rearrangements and evolution of tRNA Pseudogenes in the mitochondrial genome of the Parrotfish (Teleostei: Perciformes: Scaridae). Journal of Molecular Evolution.

[ref-44] Macey JR, Larson A, Ananjeva NB, Fang Z, Papenfuss TJ (1997). Two novel gene orders and the role of light-strand replication in rearrangement of the vertebrate mitochondrial genome. Molecular Biology & Evolution.

[ref-45] Mitchell AL, Sangrador-Vegas A, Luciani A, Madeira F, Nuka G, Salazar GA, Chang H-Y, Richardson LJ, Qureshi MA, Fraser MI, Blum M, Rawlings ND, Lopez R, El-Gebali S, Pesseat S, Yong S-Y, Potter SC, Paysan-Lafosse T, Finn RD, Marchler-Bauer A, Thanki N, Mi H, Thomas PD, Natale DA, Tosatto SCE, Necci M, Orengo C, Sillitoe I, Attwood TK, Babbitt PC, Brown SD, Bork P, Bridge A, Rivoire C, Sigrist CJA, Redaschi N, Pandurangan AP, Gough J, Haft DR, Sutton GG, Huang H, Letunic I (2018). InterPro in 2019: improving coverage, classification and access to protein sequence annotations. Nucleic Acids Research.

[ref-46] Ojala D, Montoya J, Attardi G, amp (1981). TRNA punctuation model of RNA processing in human mitochondrial. Nature.

[ref-47] Pabijan M, Spolsky C, Uzzell T, Szymura JM (2008). Comparative analysis of mitochondrial genomes in Bombina (Anura; Bombinatoridae). Journal of Molecular Evolution.

[ref-48] Pan S, Dang N, Wang J, Zheng Y, Rao D, Jiatang LI (2013). Molecular phylogeny supports the validity of polypedates impresus Yang 2008. Asian Herpetological Research.

[ref-49] Peng QL, Nie LW, Pu YG (2006). Complete mitochondrial genome of Chinese big-headed turtle, Platysternon megacephalum, with a novel gene organization in vertebrate mtDNA. Gene.

[ref-50] Perna NT, Kocher TD (1995). Patterns of nucleotide composition at fourfold degenerate sites of animal mitochondrial genomes. Journal of Molecular Evolution.

[ref-51] Ren Z, Zhu B, Ma E, Wen J, Tu T, Cao Y, Hasegawa M, Zhong Y (2009). Complete nucleotide sequence and gene arrangement of the mitochondrial genome of the crab-eating frog Fejervarya cancrivora and evolutionary implications. Gene.

[ref-52] Rest JS, Ast JC, Austin CC, Waddell PJ, Tibbetts EA, Hay JM, Mindell DP (2003). Molecular systematics of primary reptilian lineages and the tuatara mitochondrial genome. Molecular Phylogenetics & Evolution.

[ref-53] Ronquist F, Teslenko M, Mark PVD, Ayres DL, Darling A, Höhna S, Larget B, Liu L, Suchard MA, Huelsenbeck JP (2012). MrBayes 3.2: efficient Bayesian phylogenetic inference and model choice across a large model space. Systematic Biology.

[ref-54] San Mauro D, Gower DJ, Zardoya R, Wilkinson M (2006). A hotspot of gene order rearrangement by tandem duplication and random loss in the vertebrate mitochondrial genome. Molecular Biology and Evolution.

[ref-55] Sano N, Kurabayashi A, Fujii T, Yonekawa H, Sumida M (2004). Complete nucleotide sequence and gene rearrangement of the mitochondrial genome of the bell-ring frog, Buergeria buergeri (family Rhacophoridae). Genes & Genetic Systems.

[ref-56] Sano N, Kurabayashi A, Fujii T, Yonekawa H, Sumida M (2005). Complete nucleotide sequence of the mitochondrial genome of Schlegel’s tree frog Rhacophorus schlegelii (family Rhacophoridae): duplicated control regions and gene rearrangements. Genes & Genetic Systems.

[ref-57] Schubert M, Lindgreen S, Orlando L (2016). AdapterRemoval v2: rapid adapter trimming, identification, and read merging. BMC Research Notes.

[ref-58] Shao R, Barker SC, Mitani H, Aoki Y, Fukunaga M (2005). Evolution of duplicate control regions in the mitochondrial genomes of metazoa: a case study with australasian ixodes ticks. Molecular Biology & Evolution.

[ref-59] Steinauer ML, Nickol BB, Broughton R, Ortí G (2005). First sequenced mitochondrial genome from the phylum Acanthocephala (Leptorhynchoides thecatus) and its phylogenetic position within Metazoa. Journal of Molecular Evolution.

[ref-60] Suga K, Mark Welch DB, Tanaka Y, Sakakura Y, Hagiwara A (2008). Two circular chromosomes of unequal copy number make up the mitochondrial genome of the rotifer Brachionus plicatilis. Molecular Biology & Evolution.

[ref-61] Tamura K, Stecher G, Peterson D, Filipski A, Kumar S (2013). MEGA6: molecular evolutionary genetics analysis version 6.0. Molecular Biology and Evolution.

[ref-62] Ulianosilva M, Americo JA, Costa I, Schomakerbastos A, Rebelo MDF, Prosdocimi F (2015). The complete mitochondrial genome of the golden mussel Limnoperna fortunei and comparative mitogenomics of Mytilidae. Gene.

[ref-63] Voet D, Voet JG, Pratt CW (2013). Fundamentals of biochemistry: life at the molecular level.

[ref-64] Walker BJ, Abeel T, Shea T, Priest M, Abouelliel A, Sakthikumar S, Cuomo CA, Zeng Q, Wortman J, Young SK (2014). Pilon: an integrated tool for comprehensive microbial variant detection and genome assembly improvement. PLOS ONE.

[ref-65] Xia Y, Zheng Y, Miura I, Wong PB, Murphy RW, Zeng X (2014). The evolution of mitochondrial genomes in modern frogs (Neobatrachia): nonadaptive evolution of mitochondrial genome reorganization. BMC Genomics.

[ref-66] Yong HS, Song SL, Lim PE, Eamsobhana P, Tan J (2016). Complete mitochondrial genome and phylogeny of Microhyla butleri (Amphibia: Anura: Microhylidae). Biochemical Systematics & Ecology.

[ref-67] Yu D, Zhang J, Zheng R, Shao C (2012). The complete mitochondrial genome of Hoplobatrachus rugulosus (Anura: Dicroglossidae). Mitochondrial DNA.

[ref-68] Yuan S, Xia Y, Zheng Y, Zeng X (2016). Next-generation sequencing of mixed genomic DNA allows efficient assembly of rearranged mitochondrial genomes in Amolops chunganensis and Quasipaa boulengeri. PeerJ.

[ref-69] Zhang P, Liang D, Mao RL, Hillis DM, Wake DB, Cannatella DC (2013). Efficient sequencing of Anuran mtDNAs and a mitogenomic exploration of the phylogeny and evolution of frogs. Molecular Biology & Evolution.

[ref-70] Zhang JY, Zhang LP, Yu DN, Storey KB, Zheng RQ (2018). Complete mitochondrial genomes of Nanorana taihangnica and N. yunnanensis (Anura: Dicroglossidae) with novel gene arrangements and phylogenetic relationship of Dicroglossidae. BMC Evolutionary Biology.

[ref-71] Zhang P, Zhou H, Chen YQ, Liu YF, Qu LH (2005a). Mitogenomic perspectives on the origin and phylogeny of living amphibians. Systematic Biology.

[ref-72] Zhang P, Zhou H, Liang D, Liu YF, Chen YQ, Qu LH (2005b). The complete mitochondrial genome of a tree frog, Polypedates megacephalus (Amphibia: Anura: Rhacophoridae), and a novel gene organization in living amphibians. Gene.

[ref-73] Zhuang X, Cheng CHC (2010). ND6 gene lost and found: evolution of mitochondrial gene rearrangement in antarctic notothenioids. Molecular Biology & Evolution.

