# Supplementary material for: The revised complete mitogenome sequence of the tree frog Polypedatesmegacephalus (Anura, Rhacophoridae) by next-generation sequencing and phylogenetic analysis
Source: PeerJ. 2019 Aug 1;7:e7415. doi: 10.7717/peerj.7415 (PMC6679912; doi:10.7717/peerj.7415)
Supplement: Dataset S2 [file peerj-07-7415-s002.docx]

LOCUS BSeq#1 19952 bp DNA circular 21-SEP-2018

DEFINITION Polypedates megacephalus voucher 20130003 mitochondrion, complete

genome.

ACCESSION BSeq#1

VERSION

KEYWORDS .

SOURCE mitochondrion Polypedates megacephalus voucher 20130003

ORGANISM Polypedates megacephalus voucher 20130003

Unclassified.

REFERENCE 1 (bases 1 to 19952)

AUTHORS Huang,A. and Zhang,M.

TITLE Unpublished

JOURNAL unpublished

REFERENCE 2 (bases 1 to 19952)

AUTHORS Huang,A. and Zhang,M.

TITLE Direct Submission

JOURNAL Submitted (21-SEP-2018) College of Science and Technology, Sichuan

Agricultural University, #211 Huimin Road Wenjiang, Chengdu,

Sichuan 611130, P.R. China

COMMENT Bankit Comment: TAX: No, not new species/combinations; SEE

ATTACHMENT

Bankit Comment: ALT EMAIL:99709978@qq.com

Bankit Comment: TOTAL # OF SEQS:1

##Assembly-Data-START##

Assembly Method :: A5-miseq v. v20150522; SPAdes v. v3.9.0

Sequencing Technology :: Illumina

##Assembly-Data-END##

FEATURES Location/Qualifiers

source 1..19952

/organism="Polypedates megacephalus voucher 20130003"

/organelle="mitochondrion"

/mol_type="genomic DNA"

/country="China"

/collection_date="2017"

/collected_by="MingWang Zhang"

tRNA 1..71

/product="tRNA-Thr"

tRNA 72..143

/product="tRNA-Leu"

/note="codons recognized: CUN"

tRNA complement(149..217)

/product="tRNA-Pro"

tRNA 219..288

/product="tRNA-Phe"

rRNA 287..1216

/product="12S ribosomal RNA"

tRNA 1217..1285

/product="tRNA-Val"

rRNA 1286..2856

/product="16S ribosomal RNA"

tRNA 2859..2932

/product="tRNA-Leu"

/note="codons recognized: UUR"

gene 2936..3896

/gene="ND1"

CDS 2936..3896

/gene="ND1"

/note="TAA stop codon is completed by the addition of 3' A

residues to the mRNA'"

/codon_start=1

/transl_table=2

/product="NADH dehydrogenase subunit 1"

/translation="MTQMLYLTHPIMFIAPILLAVAFFTLIERKVLGYMQHRKGPNKV

GPFGLLQPIADGVKLFIKEPVQPATAAQMLFILTPTLALILAMIMWAPFPMPTPQSDI

TMSILFILAVSSLTVYTILGSGWASNSKYALIGSLRAVAQTISYEVTMALIILCSIFL

AGGFTLSIFNVTQQHLWLLLPLWPLALMWYITTLAETNRAPFDLTEGESELVSGFNVE

YASGPFALFFLAEYANILMMNTLSTILFLGAAMLMKTLSTIFLMLKASSMSIYFLWIR

ASYPRFRYDQLMHLAWKNFLPITIAATMIFISMPTSTLISPPIM"

tRNA 3897..3967

/product="tRNA-Ile"

tRNA complement(3967..4037)

/product="tRNA-Gln"

tRNA 4037..4105

/product="tRNA-Met"

gene 4106..5143

/gene="ND2"

CDS 4106..5143

/gene="ND2"

/codon_start=1

/transl_table=2

/product="NADH dehydrogenase subunit 2"

/translation="MNPLAYSMFLLSLAIGTTTTLLSHHWLLAWIGLEINTLAIIPVI

TKIPHPRAIEAATKYFLTQATASIIVLFSALMNAWSTGQWTITSALDHMTIILTLALM

MKLGLAPLHFWLPEVLQGTPLFTGLIISTWQKIAPMTLLLQLTHHINIYAATSIGLLS

IFIGGWGGINQTQMRKILAFSSIGHLGWMMLVLKFDPSLTEFNFILYIIMTTAMFLLL

TSTHAMKLLDISILWAKTPMLVAISMLTLLSLAGLPPLTGFMPKLMISLELIKQNMVI

FVTLAMLASLLALFFYLRLTYVMVMITAPNMSTTNSLWRMTIKINSLTALFNNLAIFT

IMMTPTIILFL"

tRNA 5142..5212

/product="tRNA-Trp"

tRNA complement(5213..5282)

/product="tRNA-Ala"

tRNA complement(5284..5356)

/product="tRNA-Asn"

rep_origin complement(5359..5384)

/note="L-strand origin of replication"

tRNA complement(5384..5448)

/product="tRNA-Cys"

tRNA complement(5449..5515)

/product="tRNA-Tyr"

gene 5520..7073

/gene="COI"

CDS 5520..7073

/gene="COI"

/codon_start=1

/transl_table=2

/product="cytochrome c oxidase subunit I"

/translation="MLTRWFFSTNHKDIGTLYLVFGAWAGMIGTALSLLIRAELSQPG

TLLGDDQIYNVIVTAHAFVMIFFMVMPILIGGFGNWLVPLMLGAPDMAFPRMNNMSFW

LLPPSFLLLLASSTVEAGVGTGWTVYPPLAGNLAHAGPSVDLAIFSLHLAGVSSILGA

INFITTILNMKPAALTQYQTPLFIWSVLITAVLLLLSLPVLAAGITMLLTDRNLNTTF

FDPAGGGDPVLYQHLFWFFGHPEVYILILPGFGIISHVVAFYSYKKEPFGYMGMVWAM

LSIGFLGFIVWAHHMFTTDLNVDTRAYFTSATMIIAIPTGVKVFSWLATMHGGIIKWD

AAMLWALGFIFLFTVGGLTGVVLANSSIDIVLHDTYYVVAHFHYVLSMGAVFAIMAGF

VHWFPLFTGYKLHAAWTKIQFVIMFAGVNLTFFPQHFLGLAGMPRRYSDYPDAYTLWN

TTSSIGSVISLIAVMVMIFIVWEAFSSKRVFLHSDLTSSNVEWTLGFPPAHHTFEEAT

FSITLREKG"

tRNA complement(7061..7131)

/product="tRNA-Ser"

/note="codons recognized: UCN"

tRNA 7133..7201

/product="tRNA-Asp"

gene 7202..7891

/gene="COII"

CDS 7202..7891

/gene="COII"

/codon_start=1

/transl_table=2

/product="cytochrome c oxidase subunit II"

/translation="MAQPTQFGLQDAASPIMEELIHFHDHTLMAVLLISTLVLYIIIT

LMASKLFSTNLTDAQEIEMVWTIMPAVILIVIALPSLRILYLMDEISSPDLTVKTVGH

QWYWTYEYSDFDNIGFDSYMLPTKDLDPGSFRLLEVDNRMVVPTGTLTRILVTADDVL

HSWALPSLGIKTDAIPGRLTQASILVAHPGVYYGQCSEICGANHSFMPIVVEALPIRD

FLPWILEMQDS"

tRNA 7897..7966

/product="tRNA-Lys"

gene 8829..9507

/gene="ATP6"

CDS 8829..9507

/gene="ATP6"

/note="TAA stop codon is completed by the addition of 3' A

residues to the mRNA"

/codon_start=1

/transl_table=2

/product="ATP synthase subunit 6"

/translation="MGSLFSQFTSPTLFGIHLFLIAMIFPWLFFYSPSNRWIPNRLIT

VQSLFLGTFAKHILMPTNKPAHKWALLIVTVMVFLLSMNLMGLLPYTFTPTTQLPLNL

GLAIPLWLATVLVGLRNQLSASLAHFLPEGTPTPLIPILIIIETISLLIRPIALGVRL

MANLTAGHLLIQLIATATMFMIPLSTVVSLMSFTVLLLLTILEIAVAMIQAYVFVLLF

SLYLHENT"

gene 9508..10291

/gene="COIII"

CDS 9508..10291

/gene="COIII"

/note="TAA stop codon is completed by the addition of 3' A

residues to the mRNA"

/codon_start=1

/transl_table=2

/product="cytochrome c oxidase subunit III"

/translation="MNHQAHAFHMVNPSPWPLTGASAAFLLTCGLAMWFHFNSTVTMT

LGMMLMFLTMYQWWRDVVREGTFQGHHTIPVHKGLRYGMVLFITSEIFFFMGFFWAFY

NASLAPTPDVGETWPPTGITPLNPFEVPLLNTAVLLASGVSVTWAHHSVMLGNKKNMI

QALTLTILLGIYFTALQAMEYYEAPFTLADGIYGTTFFVATGFHGLHVIIGTMFLFTC

LLRQMFNHFTTQHHFGFEGAAWYWHFVDVVWLFLFVSIYWWGF"

tRNA 10292..10359

/product="tRNA-Gly"

gene 10360..10699

/gene="ND3"

CDS 10360..10699

/gene="ND3"

/note="TAA stop codon is completed by the addition of 3' A

residues to the mRNA"

/codon_start=1

/transl_table=2

/product="NADH dehydrogenase subunit 3"

/translation="MLAFLLVTILLSVILAGASFWLATLLPDTEKLSPYECGFDPLGS

ARLPYSMRFFLVAILFLLFDLEIALLLPLPWALQLPHPLPVIMWATVILLLLTAGFLY

EWLQDGLEWAE"

tRNA 10700..10768

/product="tRNA-Arg"

gene 10769..11053

/gene="ND4L"

CDS 10769..11053

/gene="ND4L"

/codon_start=1

/transl_table=2

/product="NADH dehydrogenase subunit 4L"

/translation="MVYLLPTMYLITLIGLAAHRMHLLSALLCLEGLMLVTYIALSLC

PNYLTLAAYVFPVFLLSMSACGAALGLALMVATYRSHGNDNLITLNLLQC"

gene 11047..12409

/gene="ND4"

CDS 11047..12409

/gene="ND4"

/note="TAA stop codon is completed by the addition of 3' A

residues to the mRNA"

/codon_start=1

/transl_table=2

/product="NADH dehydrogenase subunit 4"

/translation="MLILILSWTAIFFTTALASPKNLWLITTKQSFLIAFLSLLWFVN

QNFYFSNSLFIIDHISGPLAILTCWLFPLTMLASQSKLSLEPVNRQRMYIANSTFLQL

TTLLAFSTSDLMLFFIFFEASLIPTMIIITRWGAQERRLEAGMYLAFYTMMGAVPLMI

WFIKLYVLSGSVLPVYLKTMSIMQPETTFPGLFWTMYNAAFLVKLPMYCLHLWLPKAH

VEAPIAGSMVLAGTLLKLGGYGILRTSLVLPNVFLSKTLPIMMIAIMGVLMTALLCLR

QTDLKSLIAMSSVSHMNLVIAAALISTPWSYSGATIMMIAHGLSSSALFCLANTAYER

TNSRTMIIIRGMILLFPLGWAWWLIMALFNIALPPSISFAGEFLIITSLFNWSPVTFI

FVVMNLIFTTAYTLYVLWATQRGPLPNHIKTLFPLSTREHILLYLHILPALLLITKPN

LIMF"

tRNA 12410..12478

/product="tRNA-His"

tRNA 12479..12546

/product="tRNA-Ser"

/note="codons recognized: AGY"

gene complement(12549..13040)

/gene="ND6"

CDS complement(12549..13040)

/gene="ND6"

/codon_start=1

/transl_table=2

/product="NADH dehydrogenase subunit 6"

/translation="MIAEMNLLIGLVAVASNPSPFFAALGLVWGAGAGCFVLVKSGLG

FLSLVLFLIYLGGMMVVFAYCSALVAEPYPEAWGNYTVLYYIFLYLLVLLMGVVLVGG

MGGDIKYNMGQDLILVEWWGVAELLGAGGWMLVCCGWGLLLTLFVVLEVVRGYSVGAL

RAV"

tRNA complement(13041..13109)

/product="tRNA-Glu"

gene 13114..14269

/gene="Cytb"

CDS 13114..14269

/gene="Cytb"

/note="TAA stop codon is completed by the addition of 3' A

residues to the mRNA"

/codon_start=1

/transl_table=2

/product="cytochrome b"

/translation="MTPAMRKAHPAIKIINNSFIDLPTPANLSAWWNFGSLLGVCLIA

QVATGLFLAMHYTADTSLAFSSVAHVCRDVNYGWLLRNLHANGASFFFICVYLHIGRG

LYYGSFNFKETWNIGVILLFLLMATAFVGYVLPWGQMSFWGATVITNLMSAAPYIGNT

LVQWIWGGFSVDNATLTRFFTFHFILPFAIMGASMMHLLFLHQTGSSNPTGLNSNLDK

IPFHPYFSYKDILGFIIMLTSLALLSTFNPNLLGDPDNFTPANPLVTPPHIKPEWYFL

FAYAILRSIPNKLGGVLALLFSIIVLLFVPLTNTSKQRTMAFRPLTKILFWVLIANTV

ILTWIGGQPVEDPFIIIGQLASGLYFIVFILLLPLTGVAENKLMNLPHHTM"

D-loop 14270..15843

gene 15844..17622

/gene="ND5"

CDS 15844..17622

/gene="ND5"

/codon_start=1

/transl_table=2

/product="NADH dehydrogenase subunit 5"

/translation="MAPALTIFIIMLTPLLRLGKTTFPAMAKTAIKTAFFVSLTLLSL

TSYTHWWMSTLNLSWLTLNASPIPLMIQFDTYSLLFMTVAFFVSWSIMEFSAWYMHSD

PNLETFMKYLLIFLMAMIVLVSAGNLFTLFIGWEGVGIMSYLLIGWFHGRANAATAAV

QAVLYNRIGDIGFLVIFCWALKELTLTNMQAMYSFHPPTPILMAFILAAASKSAQFGL

HPWLAAAMEGPTPVSALLHSSTMVVAGIFLMIRIHPIIAQNPHALTTCLCLGALSTFY

AAASALPQNDIKKVIAHSTSSQLGLMMVAIGINQPHLAFFHICTHAFFKAMLFLCAGL

FIHSLNDEQDIRKMGGLFNILPMTTTSFLVGSLALMGTPFLSAFYSKDAIIETMNNSF

TNSVALTLTLIATAFTAVYSVRLIYFVSLGNTRLNNPTTLINESSTMSAVTPIIRLAY

GSIFAGVMIFQLLIPNTPIIHTMSMSNKLTATVLTILAFLLAFDIIKMPTLAKWPTKK

LLDPSLYNFVIHRLSAKTALAIAGYTISFMVESFMLKTKATSIMHAQKPAMQILQQAQ

TGKIKTYLLSTTMTLMMAYAALMIGL"

D-loop 17623..19952

BASE COUNT 6020 a 4915 c 2948 g 6069 t

ORIGIN

1 gtcctggtag cttaagctaa agcgttggtc ttgtaaacca aagaatgtga acttatattt

61 tacccaggac tgcttttaat ggaaaaaagt cttccactgg ctttaggcgc caacaactct

121 tggtgcaaac ccaagtaaaa gctttaactc aaaacaaaag gaatttaacc tcttccactg

181 actcccaaag ccagtattct aattaaacta tgttttgaac tcctatagct taaacccaaa

241 gcatagtgct gaaaacgcta agatgaacca taaaaagttc taagagtaac aagtttaatc

301 ctgactttaa ccttaactat ttcttaattt acacatgcaa gtctcagcac tcccgtgaaa

361 acgcccttta gccctttaac aggataagga gctggcatca ggcacagaac ccttgcccat

421 aacgcctagt ttcaccacgc ctccaaaggt agtcagcagt gatcgacatt gtctataggc

481 gcaagcccga atcagttaaa gaaaagagag tcggcaaacc cggtgccagc cgccgcggcc

541 ataccgtgga ctcaagttaa taactaccgg cgtaaagcgt gattaaagag cccctaaatt

601 agagttgaat ggaggtttag ctgtttaaag cttacttatt aagaaaccca aaaacgaaag

661 ttactctaat taatttcttg aatacacgaa agtttgggaa caaactggga ttagataccc

721 cactatgcca taccataaat ttcacttaca cttaaaacgc cagggtacta cgagccccgc

781 ttaaaaccca aaggatttga cggtgttcca cccaactaga ggagcctgtt ctataatcga

841 caatccacga tctacctaac cattttttgc tactcagcct gtatacctcc gtcgtaagct

901 tatcatttga atgagaaaaa gtaagcacaa ggactaaaag ccaaaacgtc aggtcaaggt

961 gcagcttata aaatggaaga aatgagctac aatttctaat ttagaacaca cgaattactg

1021 catgaaacaa agtcatgaag gtggatttag cagtaagtaa ggaatagaga gccttactta

1081 atttggccct ggaacgtgta caaaccgccc gtcaccctct tcgatagctt tttactagtt

1141 aaataaccta tttgccgcgc cttagaagag gcaagtcgta acatggtaag tatactggaa

1201 agtgtacttg gaataccaaa atgtagctta acaaaagccc cccgcttaca ccgggaaaat

1261 gtccgtgcaa cccagaccat tttgagccct aaatttagcc ctaaattatt ttataaactt

1321 cttatacgca aagttattaa ataaaacatt ttaactcctt aagtaaaggc gattgaaaaa

1381 gctctaagcg ctatagaaat agtaccgcaa gggaaaaatg aaataataat gaaacaacta

1441 aagctactca cagcagagaa ttcacctcgt accttttgca tcatggccta gcaagactta

1501 ataagcaaaa tgaaatttaa gttttaaacc ccgaacctag gcgagctact ttaaaacagc

1561 ctattgggcc aacccgtctc tgtagcaata gagtgggatg atttttaagt agtagtgaaa

1621 agcctaccga gcctagaaat agctggttat tcatgaaaag aatataagtt ctgccttaag

1681 cctccctcaa tgaacctcaa caacattcaa ggcttaagag ttatttaaat aaggaacagc

1741 ttatttaagc caggacacaa cctaagcagc agggtaaagt cttaacacta ccaagtgggc

1801 ctgaaagcag ccatctttta aaacgcgtca aagcgtaacc ttaattccca aataccatta

1861 atcttcatga acccctaatt tataatgaat gactccatat ttatatggaa gacaccatgc

1921 taggattagt aacaagaatt tataattctc caaaatgcaa acataaacca acatggatag

1981 accattggta gttaccgaac ctaaattcac tgcaataaca cccacaagaa aactctgcag

2041 tacttactcg ttaacctaac actagcgcat tacaggaaag atttaaaaaa agagaaggaa

2101 ttcggcaaat cacggccccg cctgtttacc aaaaacatcg cctcttgact tataataaga

2161 ggtccagcct gcccagtgaa tttttaacgg ccgcggtatc ctaaccgcgc gaaggtagca

2221 taatcacttg ttctttaaat ggggactcgt atcaacggca tcacgagggt tttactgtct

2281 cctctttttg atcagtgaaa ttgatcttcc cgtgaagaag cgggaataat actataagac

2341 gagaagaccc catggagctt aaaaccaaat aacaacttaa ccactcaccc ccctataaat

2401 ttaagccctg ttataaggtt ttcggttggg gtgaccgcgg agtaaagctc atcctccacg

2461 acggaaagaa cacactcttt atttaagaac cacaattcta gaaattagta tactaacgtt

2521 tactgacccg ataatcgatc aacggaccaa gttaccctgg ggataacagc gcaatctact

2581 tcaagagctc atatcgacaa gtaggcttac gacctcgatg ttggatcagg gtatcccggt

2641 ggtgcagccg ctaccgaagg ttcgtttgtt caacgattaa aaccctacgt gatctgagtt

2701 cagaccggag caatccaggt cggtttctat ctataaagag ttacttctag tacgaaagga

2761 ccgaagtaac gtggccaatg cacccccaag ccacaaattc tactaatgat tacaacttaa

2821 ttagcaagta tctatttaat tattaaagtc aatgatttgt tagcgtggca gaacatggct

2881 atgcgaaaga cctaagccct ttacactagg ggttcaagtc ccctcgttaa cttaaatgac

2941 tcaaatactt tacctcaccc acccaattat atttattgcc ccaattctac ttgcagtggc

3001 attttttaca ttaattgaac gaaaagtatt aggctatata caacaccgca aaggccctaa

3061 taaagtaggc ccatttggac tcctgcaacc aattgcagac ggagttaaac tatttattaa

3121 agagcctgtc cagccagcta cggcagcgca aatattgttc atcttaaccc ctacgctagc

3181 tcttattcta gcaataatta tatgagcacc atttccgata cccacgcctc agtcagatat

3241 tactataagt atcctattta tcctcgctgt ctcaagcctc actgtttata ctattctcgg

3301 ctccggatga gcatcaaatt ctaaatatgc ccttattggt tctttacgcg ctgtagcaca

3361 gaccatttct tatgaagtca caatagccct aattatcctt tgttcaatct tcttggctgg

3421 cggcttcaca ctgtccatct ttaatgttac acaacaacac ttatgattac tcctcccctt

3481 atgacctcta gcacttatat gatatattac tacactagcc gagacaaacc gagccccatt

3541 tgatctaact gaaggagaat cagaacttgt ctcaggcttt aatgttgaat acgcaagcgg

3601 cccgttcgcc ttattcttcc tcgccgaata tgctaatatc ttaataataa acactttatc

3661 aactatttta ttcctcggag ccgcaatatt aataaaaacg ctctcaacaa tttttctcat

3721 gttaaaagca tcatcaatat ccatttattt tttatgaatt cgtgcctcat atccacgatt

3781 ccgttatgac caacttatac atcttgcctg gaagaacttc ctgcctatta caatcgccgc

3841 tacaataatc tttatctcta taccaacctc aactttgatt tcccccccta ttatatggaa

3901 gcgtgcccga aagataagga cctccttgat agggaggcta atatgggttc aaaccccatc

3961 acttccttaa agagatagga gttgaaccta cgtctaaggg atcaaaaccc ttcgctattc

4021 caccttgctt ctctttagta agatcagcta aaaaagcttt tgggcccata ccccaacaat

4081 gttggttaaa ctccttctct tactaattaa ccctctagct tactctatat ttttacttag

4141 cctcgcaatt ggcacaacaa ctaccctgtt aagccaccac tgactgctcg cttgaatcgg

4201 cctagaaatt aatacactag ctattatccc tgtcattacc aaaatccctc atccacgggc

4261 tattgaggcc gccacgaaat atttcttaac acaagcaacg gcttctatta ttgttctatt

4321 ttcagcacta ataaacgcat gaagcacagg acaatgaacc atcacctctg ccctcgacca

4381 tataacaatt atcttgacct tagctctgat aataaaacta ggattagctc cactacactt

4441 ctgactcccc gaagtactcc aaggcacccc attatttacc ggactaatta tctcaacgtg

4501 acaaaaaatc gccccaataa cattgttact tcaacttaca caccatatta atatttatgc

4561 cgccacttca attggcttac tgtcaatttt tattggcgga tgaggaggaa ttaatcaaac

4621 acaaatacgg aaaattctag cattctcatc aattggccac ctcggctgaa taatattagt

4681 tttaaaattt gacccctctt taacagagtt caattttatc ttatatatta ttataacaac

4741 agcaatattt cttttactga cctcaacaca cgctataaaa ttacttgaca tctccatttt

4801 atgggcgaaa acaccaatat tagttgcaat ttcaatgctt acattactct ccttagcagg

4861 cctcccacct cttactggct ttatacctaa actaataatc tctttagaac taattaaaca

4921 gaacatagtt atttttgtaa cgctcgctat attagcctca ctccttgccc tattcttcta

4981 cctacgcctc acctacgtca tagttataat taccgcacca aatatatcta ccaccaatag

5041 cctatgacgc ataaccatca aaattaattc actgacggca ctatttaaca atcttgcaat

5101 ctttactatc ataataacac caacaattat tcttttttta tagaaattta gggtaatgcc

5161 agcccgaagg ccttcaaagc cttaagcaag agtttaaatc tcttaatttc tgtaggactt

5221 gtaggatact aacctacatt tactgaatgc aactcaaact ttttaaatta aaataaagcc

5281 cttctagaaa gacgggcctt gatcccgtaa tattttagtt aacagctaaa cactctatcc

5341 agcgagcttc attctacttc tcccgtctac agcgaaacgg gagaagcccc ggcagaactg

5401 cttctgcttc ttgcggtttg cacccgcacg tgtaacaccc cgcgggcctg gtaaagagag

5461 ggtttacacc tctgtccttg gggctacaat ccaccgccta ctcggccact ttacctgtga

5521 tattaacccg ctgatttttc tctactaacc acaaggacat tggtacactg tacttggtct

5581 ttggtgcctg agccggcatg attggcactg cccttagcct gcttatccgc gccgaactat

5641 cccaacccgg caccctgctc ggcgatgatc aaatctataa tgtcattgtt actgcccatg

5701 catttgtaat aattttcttt atggttatac caattcttat tgggggattc ggtaactgac

5761 ttgtcccact aatacttggc gccccagata tagctttccc acgtataaat aacataagtt

5821 tctggctcct gcccccatca tttctccttc ttcttgcctc ttcgactgtc gaggctggtg

5881 tcggaactgg ctgaactgta tatcccccct tagcagggaa cctggcccat gcaggcccgt

5941 cagttgacct agctattttt tcccttcacc tagctggggt gtcgtcaatt ttaggtgcca

6001 ttaactttat tacaaccatt ttaaatataa agccagcggc actaacacag taccaaaccc

6061 ccttattcat ctgatccgtt cttattactg ctgtattgct tcttctttct ctccctgtgt

6121 tagccgcagg aatcacgata cttctcaccg accgcaacct taacacaact ttctttgacc

6181 ccgcgggagg gggggaccca gttctttatc aacatctatt ttgattcttt ggacaccccg

6241 aggtctatat tcttattctt ccgggattcg gtattatttc gcatgttgtt gcgttctatt

6301 cttataagaa agagccattt gggtatatgg ggatggtttg agctatgtta tcaattgggt

6361 tcctcggctt tattgtatgg gctcaccata tatttactac agaccttaat gttgacacgc

6421 gcgcatactt tacctcggca acaataatta ttgctattcc aactggcgtg aaggtattta

6481 gctgactagc tacaatgcac ggaggaatta ttaaatgaga cgcagcaatg ctctgggccc

6541 tgggctttat ttttcttttt accgttggtg gcctcacagg agttgtactt gctaactcct

6601 caattgatat tgtgctccat gatacatatt atgttgttgc acacttccac tatgtcctgt

6661 caataggcgc agtttttgcc attatagcag gcttcgtaca ctgattcccc ttatttaccg

6721 gctacaaact ccacgctgcc tgaacaaaaa ttcaatttgt tattatgttt gctggtgtaa

6781 accttacatt ttttcctcag cattttctag ggctcgccgg tataccacga cggtactcag

6841 actacccaga cgcatataca ttatgaaata ccacctcctc cattggctct gtaatctcac

6901 taattgctgt tatggtaata atttttattg tctgagaagc attttcatct aaacgtgttt

6961 tccttcactc agatctaacg tcttcaaacg tagaatgaac actgggcttc ccccctgctc

7021 atcacacatt tgaagaagca acattctcta ttacactgcg cgagaaagga aggagttgaa

7081 ccctcttgac ctggtttcaa gccaggcaca taacccgtct gtcactttct tggaggtact

7141 aattaaagta taatattacc ttgtcaaggt gaatttacaa gtgagaccct tgtgttcctc

7201 tatagcacaa cccacacaat ttggccttca agacgccgca tcccctatta tggaagagtt

7261 aattcacttc cacgaccaca cgcttatagc agttctttta attagcacat tagtgttata

7321 cattattatt acgctcatag catctaaatt atttagtact aatttaacag acgcacaaga

7381 aattgaaata gtatgaacta ttataccagc agtgatctta atcgtcattg cacttccatc

7441 attgcgaatc ctttacttaa tagatgagat ttcttcgcct gacttgactg ttaaaaccgt

7501 cggccatcaa tggtactgaa cctatgaata ttcagatttt gataacattg gatttgactc

7561 gtatatacta cccactaaag acctcgaccc cggctcattc cgcctactag aggtggataa

7621 tcgaatagta gtccctacag gaacactgac gcgaatttta gttactgcag atgatgtact

7681 acactcctga gcactcccat cattaggaat taaaactgac gccatcccag ggcgactcac

7741 tcaagcatct atcctagtgg cccacccagg cgtatactat ggccagtgtt cagaaatttg

7801 tggcgctaac catagcttta taccaattgt agttgaagcc ctaccgatcc gtgacttttt

7861 accatgaatt ttagaaatac aggactctta acttctcatt aagaagctat atggtacgcg

7921 acggcctttt aagctgtaga taggtgattc caaccaccct taatgaatgt ccgcactaaa

7981 gattatcggt tccattacct taattatttt aggtaccctc ttttccctat tgcttactaa

8041 actgctgagc aactacttta tatcccgcat agtcctacca aaaggctcac gccctctggg

8101 gaaatttaca cgactatgac tatgattttc aatacgtcca tgaaaacgac tatgaacaca

8161 agtatacctg tgaatcataa tacgcccgtg gatacgtata tttattcaac tgggaacatg

8221 attctgattc cgcctacgag cctgactaat atcacaccaa tgagtgcggc tctgagtatg

8281 agcgtcaaaa aaatcatgaa cacagcttcg aaagtggcaa gtgcaacaat caaaacgaat

8341 atgaaaatga ctatgaaagc aaataaaaaa ataattctat ttgcccaggc ctcgttgacc

8401 ccacttaata ccctcttatc cactcggagc cctgggctcc gaactattac acccctatct

8461 gacacacagc agccctaaag ttgcaactga ttattcacca agcggggaac ccgcttttta

8521 tcaatatagc ccaccagatt atctgtataa tattaacacc ggctcagaac cacattatgt

8581 gttaatatga ttattccaca actctaacca ccccattaag aaactaatgc gtagcagttt

8641 ttcggctgca gctgggtgac ttctcgccac ccttaatgaa tggtcaaaac acttttcttt

8701 ttcattcacg tagccctatg agctattctt attattgcac ttgggacaat gtttaaaaac

8761 tatacactgt accgcacaat atcaccacta aacccccatc cgcgccgggc gcggacacca

8821 tggccttgat agggagcctg ttcagtcaat ttacatcacc aaccctattt ggtattcact

8881 tattccttat tgcaataatt tttccatggc tttttttcta ctccccatct aaccgctgga

8941 ttccaaaccg cctcatcacc gttcaatcac tgtttttagg caccttcgct aaacacattc

9001 ttatgccaac aaataaaccc gcccacaaat gagcactttt aatcgtaacc gtaatagtct

9061 ttctacttag tataaaccta ataggcttac tcccgtacac atttacccct acaacccaac

9121 tgccactaaa tttaggcttg gctatcccat tatgacttgc aactgtactc gtcggacttc

9181 gaaaccaact ttccgcatcc ttagcacatt ttctaccaga aggcacaccc acccctctaa

9241 ttcctattct aattattatt gaaacaatca gtttattaat ccgcccaatt gccttaggcg

9301 tacgcctaat agcaaacttg accgctggac atttacttat tcaactcatt gctacagcca

9361 ctatatttat gatcccactt tcaactgttg tttcgctaat gtcatttaca gttctactcc

9421 tgctaactat cctagagatt gcagtggcca taatccaagc ctatgtattt gtgcttttat

9481 ttagccttta cttacatgag aacacttatg aaccaccaag cacatgcttt tcacatagta

9541 aaccccagcc catgacccct aaccggcgcc tcagccgctt tcttgttaac ttgtggccta

9601 gccatatgat tccactttaa ctcaactgtc acaataacac tgggcataat acttatattt

9661 ttgacaatat atcaatgatg acgcgatgta gtacgcgaag gtacatttca aggtcaccac

9721 actattccag ttcataaagg tctccggtac ggaatagttt tatttattac ctcagaaatt

9781 tttttcttta tgggcttctt ctgagccttt tacaacgcca gccttgcccc aacccccgac

9841 gtaggcgaaa cgtgaccacc aacaggaatc accccactta acccttttga agtccctctt

9901 cttaatactg cggtcctatt agcctctggg gtgtccgtca cctgagcaca tcatagtgtt

9961 atactaggca ataaaaaaaa tataatccaa gcactaaccc tgacaatcct actaggaatc

10021 tattttactg ccctgcaagc gatggagtat tacgaagcgc cgtttacact tgctgatggc

10081 atttatggta caacattttt tgttgcaaca ggcttccacg gattacacgt tatcatcggg

10141 actatatttt tatttacatg cctattacgc caaatattta atcactttac aacccaacac

10201 cattttgggt ttgaaggcgc agcatgatat tgacattttg ttgacgtagt ttgactattc

10261 ctgttcgttt caatttactg atgaggtttt tattttttta gtataatagt acaaatgatt

10321 tccactcatt aagttctggt ttaaccccag gaaaaaataa tgcttgcatt cttgctagta

10381 acaattttac tgtcagttat tttagctggc gccagcttct ggctggcaac tctgctccca

10441 gacacagaaa aactctcacc ctacgaatgt ggatttgacc cgctgggctc agcccggtta

10501 ccatattcca tacgcttctt ccttgtagct attctatttt tgctttttga cttagaaatt

10561 gctcttctac taccactacc gtgagccctc caactcccac acccgcttcc agtcattatg

10621 tgagccacag taattcttct attattaacc gccgggtttt tgtacgaatg acttcaagac

10681 ggcctagaat gggcagaatg aggagctagt ctaaaaaaga cacctgattt cgactcagac

10741 aattatggtt taaccccata gcacctttat ggtttacctc ttgcccacta tgtatctaat

10801 tactcttatt ggcctagcgg ctcaccgaat acacttactt tcggccctct tgtgcctgga

10861 ggggcttatg ttagtcacct atattgccct aagcctttgc ccaaactatt taactttagc

10921 cgcatacgtt tttccagttt ttcttctttc gatatccgcc tgcggggcag ctttaggact

10981 tgcacttata gtagccacat atcgatccca tggtaatgac aaccttatta ccttaaacct

11041 acttcagtgc taatcttaat cctctcctga actgcaatct tttttaccac tgcgcttgcc

11101 tcacccaaaa acttatgact gatcacaacc aagcaaagtt ttttaattgc atttttatca

11161 ttactatggt ttgttaacca aaatttttat ttttcgaatt cgttgtttat tattgatcac

11221 atttctggcc cgttagcaat tttaacatgc tgactcttcc ccctgacaat actagcgagc

11281 caaagcaaac tcagcctaga gccagttaat cgtcaacgta tgtatatcgc taacagtaca

11341 ttccttcagc ttacaacact tctagctttt tctacatctg atttaatact cttcttcatt

11401 ttctttgaag catcattaat tcctactata attattatta cccgctgagg cgctcaagag

11461 cggcgcctag aggcaggcat atatctagca ttctatacta taataggcgc cgtaccacta

11521 atgatttgat ttattaaact atatgtccta tctggctcag tcttaccagt ctatttaaaa

11581 accatatcaa ttatacaacc agaaacaact tttccaggac tgttttgaac gatatataat

11641 gccgcatttt tagtaaaact acctatatat tgtctccact tatgactccc gaaagcccat

11701 gttgaagccc caattgcagg ctctatagtt ctagcaggca ctcttcttaa gcttgggggc

11761 tacggcatct tacgcacatc ccttgttttg cctaatgttt tcttaagcaa gacccttcca

11821 attataataa ttgctattat aggagtttta ataacagccc tgctctgcct acgccaaaca

11881 gatctaaagt cgctaattgc catgtcctca gttagccata taaacctagt gattgccgca

11941 gcattaattt ccacaccatg aagctactca ggggcaacaa tcatgataat tgcccacggg

12001 ctctcatcat cagcactatt ctgcctggca aacacagcat atgagcgaac aaattcacgc

12061 acaatgatta ttattcgagg gataatttta cttttcccac taggttgagc atgatgacta

12121 attatagccc tctttaatat tgctctccca ccttcaatta gctttgcagg agaattccta

12181 attattacct ccctatttaa ctgatcccca gttaccttta tctttgtcgt gataaattta

12241 atttttacta ccgcatacac cttatatgtt ttatgggcca cccaacgcgg acccttacca

12301 aatcacatta aaacgctttt ccctttaagc acgcgcgagc atatcctcct ctatcttcat

12361 attttaccag ctcttcttct aattaccaaa ccaaatttaa ttatgttttg tggacatagt

12421 ttaactaaaa cattaggctg tggctctaac aatgaagact taccctcttc tgtccaccga

12481 gtatggttgg agctagtgag tgttgctaac tacttattac cgcggttcaa ttccgcggct

12541 cactcgagcc tcactgcacg caaagcccca acactatatc cccgcacaac ctctaaaact

12601 acgaacaacg taagtaacag cccccaccca cagcaaacta atattcagcc ccccgctccc

12661 aataattcag caacgcccca tcattcaacc aaaattaaat cttgacctat attatactta

12721 atatcacccc ccatcccccc caccaaaact acacctatca acaacactaa caggtacaaa

12781 aaaatataat aaagcacagt ataattaccc cacgcctcgg ggtagggctc agcaaccaga

12841 gctgaacagt aagcaaacac aactattatt ccccctaaat aaattaaaaa caacactaat

12901 gacaaaaacc caagcccact tttaaccaaa acaaaacagc cagcgcccgc acctcacact

12961 aaacccaaag cagcaaaaaa cggagacgga ttggacgcca cagccaccaa cccaatcagt

13021 aaattcatct cggcaatcat ggttcctact aggggtttaa cctagaccta tagtccgaaa

13081 aactaccgct gatattcaac tacaagaacc cttatgaccc ccgcaatacg taaagctcac

13141 cctgccatca aaattattaa taactcattt attgatcttc caactcctgc caacttatct

13201 gcctgatgaa actttggatc cttattaggg gtatgcctca tcgctcaagt tgcaacaggt

13261 ttatttctag caatacacta cactgcagac acatcgcttg cattttcttc tgttgcccac

13321 gtatgtcgag acgtaaatta tggctgactg ctccgtaatc tccacgctaa cggcgcatca

13381 ttcttcttta tctgcgttta tctacacatt ggccgaggcc tatactatgg gtcattcaac

13441 tttaaagaga cctgaaacat cggtgttatt ctcctatttt tactaatggc taccgccttt

13501 gtaggctatg ttctcccatg aggacaaata tcgttttgag gcgccacggt gattacaaac

13561 ttaatgtcag cagccccata tattggcaat accttagttc aatgaatctg aggcggcttc

13621 tcagttgata atgcaacatt aacacgattc tttactttcc acttcatttt accattcgca

13681 attataggtg ctagcataat acatcttctt ttccttcacc aaacaggctc atcaaaccca

13741 acagggctaa attcaaacct agacaaaatt ccatttcatc catacttttc ctacaaggac

13801 atcttaggct ttatcattat actaacctca ctagccctat tgtccacttt taacccaaac

13861 cttcttggcg acccagacaa ctttacccca gccaaccctt tagttacacc accccacatc

13921 aaacctgaat gatacttcct ctttgcctat gcaatcctac gctcaattcc taataaacta

13981 ggaggagtcc tagcactttt attttctatc attgttcttc tctttgtacc cttaactaat

14041 acatctaaac aacgaactat ggctttccgc ccattaacaa aaattctgtt ctgagtctta

14101 atcgccaaca cagtaattct gacatgaatc ggaggccaac cagtagaaga cccatttatt

14161 attattggtc aactagcctc agggctctac ttcattgttt ttattcttct gctcccacta

14221 acaggcgtag cagaaaacaa gctcataaat ttaccccatc atactatgta taataagcat

14281 taatttattt accccatcat actatgtata ataagcatta atttatttac cccatcatac

14341 tatgtataat aagcattaat ttatttaccc catcatacta tgtataataa gcattaattt

14401 atttacccca tcatactatg tataataagc attaatttat ttaccccatc atactatgta

14461 taataagcat taatttattt accccatcat actatgtata ataagcatta atttatttac

14521 cgtacaattt ataattttta agtgaacttc agaaattaac ataaaactat ttagaacaat

14581 aaatgaatga ttttcaatta ctatagaaga atacattaat ttatttaaca catgcttatc

14641 attaccatta agggcggatt cttaataaac aactgattaa taccactaaa ctgaataaat

14701 gacccaaaat gattgccaga gccttaacta tgtctacttt atcggacctt cccttgctta

14761 agtatatgca tatcatatcc aaattttaat ccacctcttt atccacagcc cacaagaaca

14821 aattaaaatg tatgaataat aaatggtctt tattatttta caccctcaag cataacacac

14881 ctgctttaac acaatattga atgtatcatt cattataaca aggtaagacc ttcacttgcg

14941 ttagatcatg aatattccag tcaatttaat ataataatta actataatgg aatattacta

15001 ataacaataa ctaattgcgt ttaagaaaaa gagggctctt tttcttcaga ataatcattc

15061 atcacctatt attataatcc atgaatatta tctaccaatt atctgcatat tcggacataa

15121 cgtacatcca ccaaacctaa attagcaagt cctcaaccca gtaattatat tatagacctc

15181 tcgcagtgca ctctaccggg atcaattaat aagccgaccc cgtctcactt ttcatgaggc

15241 ttctgacgga actgaatcta tggacccatt aataaatcta ccctactgaa gctgttttaa

15301 aggctttagc agctggtgag aaaaggacct ttaatataag ctcaggccac ttaatgaagt

15361 cagggtcggc ccagactatt ttgggttttt ggcattcatc agcaaggtca gggtggtttc

15421 actctgagcg tcatcgccaa tatttcacta caaggcaccc actcagcagt gttcacctac

15481 ccttcccatg gacaacaaaa agcaggcatt taaattaggg tgggacaaag agctataatt

15541 gatgtccccc taatctatgg tatcccccgg gcatacctgc atgcttgtaa gacatatttt

15601 taacgatcta gcgaaatccc ctataccctt atttcccccc cctttgcact ttttttaaac

15661 ttttttcatt aaaattttac aaaatttaaa aatttttccc gctacccccc ccttaccccc

15721 cccatgcatt attctaccag cgactcctac tacccccccc gggattaaga actgttagat

15781 tttctggaag gtccacagag caatgctgct tctgagtata tacctgaatt ttccccatca

15841 ctaatggccc ccgcacttac tatttttatt attatactca cccctctatt acgacttgga

15901 aagacaacat ttcctgctat agctaaaaca gcaattaaaa cagccttttt tgtgtcatta

15961 acacttctct ccttaacgtc ctacacgcac tgatgaatat ctaccctaaa cctatcatgg

16021 ctaacactaa atgcctcccc catccccctc atgattcaat ttgacaccta ctccctccta

16081 ttcataactg tggcattctt cgtatcatga agtattatag aattttcagc atgatatatg

16141 cactccgacc ctaacctcga aacctttata aagtatctcc taattttcct tatagcaata

16201 attgtccttg tctcagcagg taacctattt actctattta tcggctgaga aggcgtagga

16261 atcatatcat atttacttat tggctgattt cacggtcgcg ctaacgccgc gaccgcagcc

16321 gtacaggcag tcttatacaa ccgaattgga gacattggat ttttagtaat cttttgctgg

16381 gcacttaaag aacttacact aactaatata caagcaatat attcattcca cccccccaca

16441 cctattctca tagccttcat ccttgctgcc gcaagcaaat ctgcccaatt tggccttcac

16501 ccatgactag cagcagcaat agaaggccct acaccagtct ctgccctact tcattctagt

16561 acaatagtcg tagccggaat ctttctaata atccgaattc accctattat tgcccagaac

16621 ccgcatgcac ttacaacctg cctatgctta ggtgctctat ccaccttcta cgccgctgca

16681 tcagcattac ctcaaaatga tatcaaaaaa gtcattgccc actcaacatc aagtcaattg

16741 ggcctaataa tagtagctat tggcatcaac cagccccacc tcgcattttt ccatatttgc

16801 acacacgcat tctttaaggc catattattc ttatgcgccg gcctcttcat tcactccctg

16861 aatgatgaac aagacattcg gaaaatgggg ggactcttca acattctacc aataacaact

16921 acttcatttt tagttggatc acttgctcta ataggtacac cgttcttatc tgctttttac

16981 tccaaagatg ctatcatcga aactataaac aactccttca ctaactccgt cgcacttacc

17041 ctgacactaa tcgcaaccgc atttacagca gtatactctg tccgactaat ttacttcgta

17101 tcgctcggca acacccgcct aaataatccc accacactta ttaatgaatc atcaaccata

17161 tccgctgtaa ccccaattat tcgcctggcc tacggcagca tctttgcagg agtaataatt

17221 ttccagctac tcatccccaa tacacctatt atccacacaa tatcaatatc caataaactt

17281 actgccacag tactaacgat tctggcattc cttcttgcct tcgatatcat caaaataccc

17341 accttagcaa aatgacccac aaaaaaactt ctagacccaa gcctttataa cttcgtaatt

17401 catcgactca gtgcaaaaac tgctctcgcc attgccggct acacaatctc atttatagtt

17461 gaatccttca tacttaaaac caaagcaaca agcattatac acgcacaaaa acccgcaatg

17521 caaatcttac agcaagcaca aacaggaaaa atcaaaacat acctattgtc aacaaccata

17581 acattaataa tggcctacgc agcacttata attggcctat aataagcatt aatttattta

17641 ccccatcata ctatgtataa taagcattaa tttatttacc ccatcatact atgtataata

17701 agcattaatt tatttacccc atcatactat gtataataag cattaattta tttaccccat

17761 catactatgt ataataagca ttaatttatt taccccatca tactatgtat aataagcatt

17821 aatttattta ccccatcata ctatgtataa taagcattaa tttatttacc gtacaattta

17881 taatttttaa gtgaacttca gaaattaaca taaaactatt tagaacaata aatgaatgat

17941 tttcaattac tatagaagaa tacattaatt tatttaacac atgcttatca ttaccattaa

18001 gggcggattc ttaataaaca actgattaat accactaaac tgaataaatg acccaaaatg

18061 attgccagag ccttaactat gtctacttta tcggaccttc ccttgcttaa gtatatgcat

18121 atcatatcca aattttaatc cacctcttta tccacagccc acaagaacaa attaaagtgt

18181 atgaataata aatggtcttt attattttac accctcaagc ataacacacc tgctttaaca

18241 caatattgaa tgtatcattc attataacaa agtaagacct tcacttgcgt tagatcatga

18301 atattccagt caatttaata taataattaa ctataatgga atattactaa taacaataac

18361 taattgcgtt taagaaaaag agggctcttt ttcttcagaa taatcattca tcacctatta

18421 ttataatcca tgaatattat ctaccaatta tctgcatatt cggacataac gtacatccac

18481 caaacctaaa ttagcaagtc ctcaacccag taattatatt atagacctct cgcagtgcac

18541 tctaccggga tcaattaata agccgacccc gtctcacttt tcatgaggct tctgacggaa

18601 ctgaatctat ggacccatta ataaatctac cctactgaag ctgttttaaa ggctttagca

18661 gctggtgaga aaaggacctt taatataagc tcaggccact taatgaagtc agggtcggcc

18721 cagactattt tgggtttttg gcattcatca gcaaggtcag ggtggtttca ctctgagcgt

18781 catcgccaat atttcactac aaggcaccca ctcagcagtg ttcacctacc cttcccatgg

18841 acaacaaaaa gcaggcattt aaattagggt gggacaaaga gctataattg atgtccccct

18901 aatctatggt atcccccggg catacctgca tgcttgtaag acatattttt aacgatctag

18961 cgaaatcccc tataccctta tttccccccc ctttgcactt tttttaaact tttttcatta

19021 aaattttaca aaatttaaaa atttttcccg ctaccccccc cttacccccc ccatgcatta

19081 ttctaccagc gactcctact accccccccg ggattaagaa ctgttagatt ttctggaagg

19141 tccacagagc aatgctgctt ctgagtatat acctgaattt tttccccccc atattattat

19201 tatgcaattt caatagatat gtagactcat cttatgaaat taatcaattt atattgatga

19261 gtttaactac tctccttgat ttcctgcgta aatatattat tatgcaattt cagtagatat

19321 gtaaactcat cttatgaaat taatcaattt atattgatga gtttaactac tctccttgat

19381 ttcctgcgta aatatattat tatgcaattt cagtagatat gtaaactcat cctatgaaat

19441 taatcaattt atattgatga gtttaactac tctccttgat ttcctgcgta aatatattat

19501 tatgcaattt caatagatat gtagactcat cttatgaaat taatcaattt atattgatga

19561 gtttaactac tctccttgat ttcctgcgta aatatattat tatgcaattt cagtagatat

19621 gtaaactcat cttatgaaat taatcaattt atattgatga gtttaactac tctccttgat

19681 ttcctgcgta aatatattat tatgcaattt caatagatat gtagactcat cttatgaaat

19741 taatcaattt atattgatga gtttaactac tctccttgat ttcctgcgta aatatattat

19801 tatgcaattt caatagatat gtagactcat cttatgaaat taatcaattt atattgatga

19861 gtttaactac tctccttgat ttcctgcgta aatatattat tatgcaattt cagtagatat

19921 gtaaactcat cctatgaaat taatcaattt at

//
