# Supplementary figures and images for: The revised complete mitogenome sequence of the tree frog Polypedatesmegacephalus (Anura, Rhacophoridae) by next-generation sequencing and phylogenetic analysis
Source: PeerJ. 2019 Aug 1;7:e7415. doi: 10.7717/peerj.7415 (PMC6679912; doi:10.7717/peerj.7415)

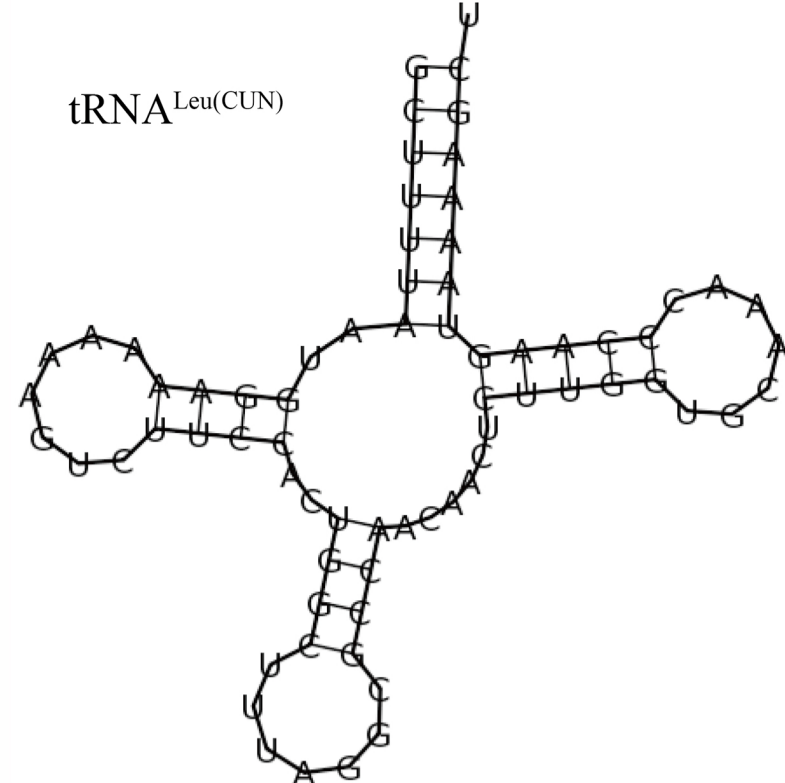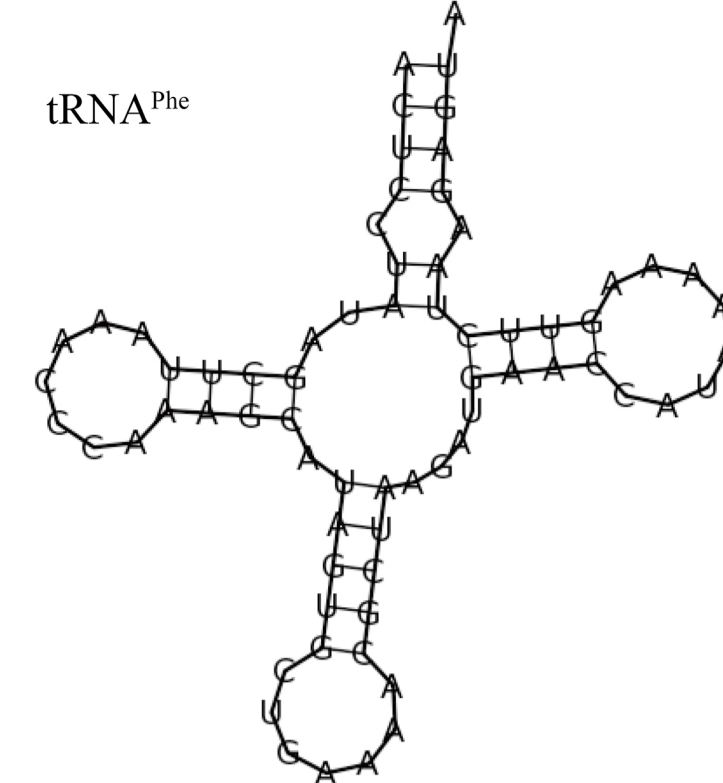

tRNA<sup>Val</sup>

tRNA<sup>Leu</sup>(UUR)

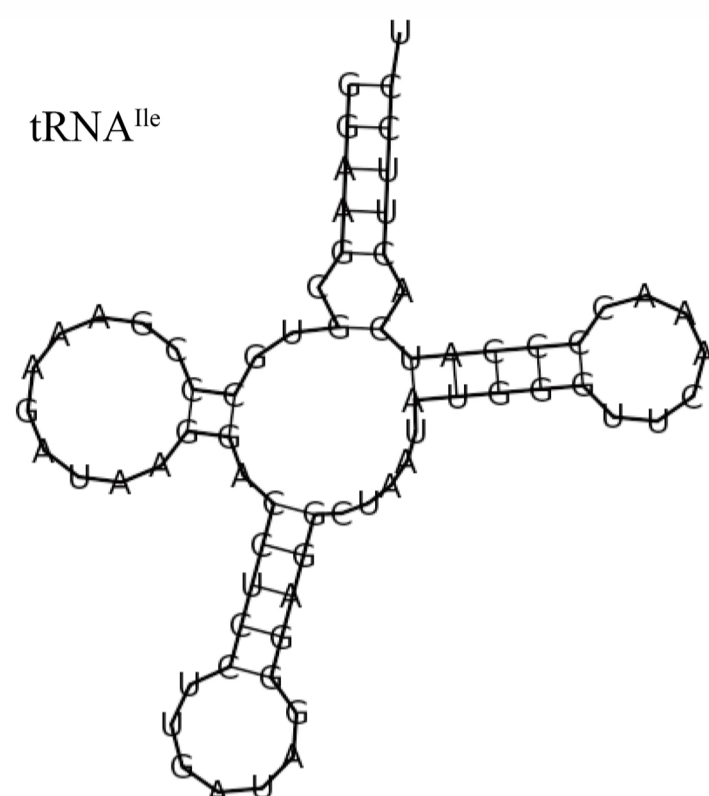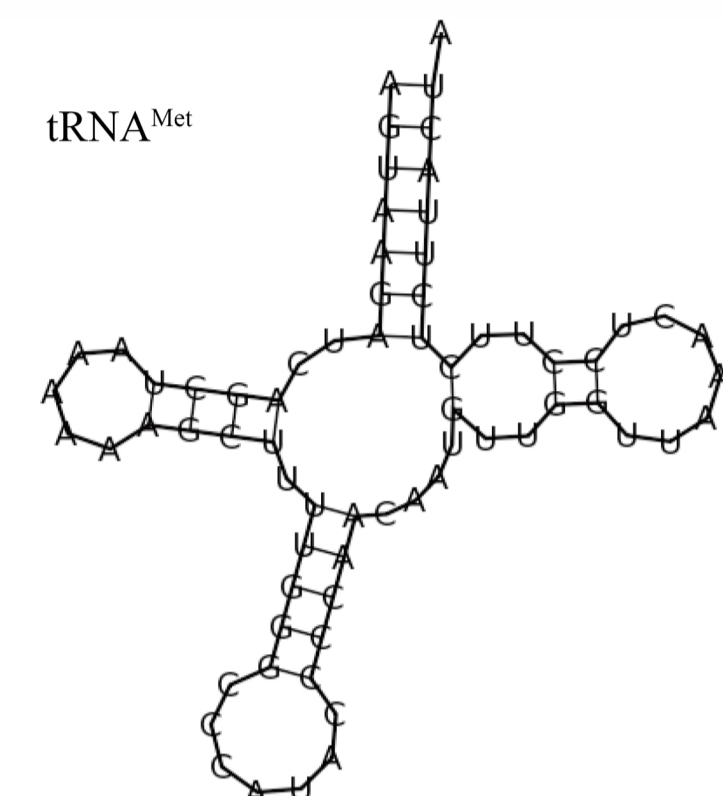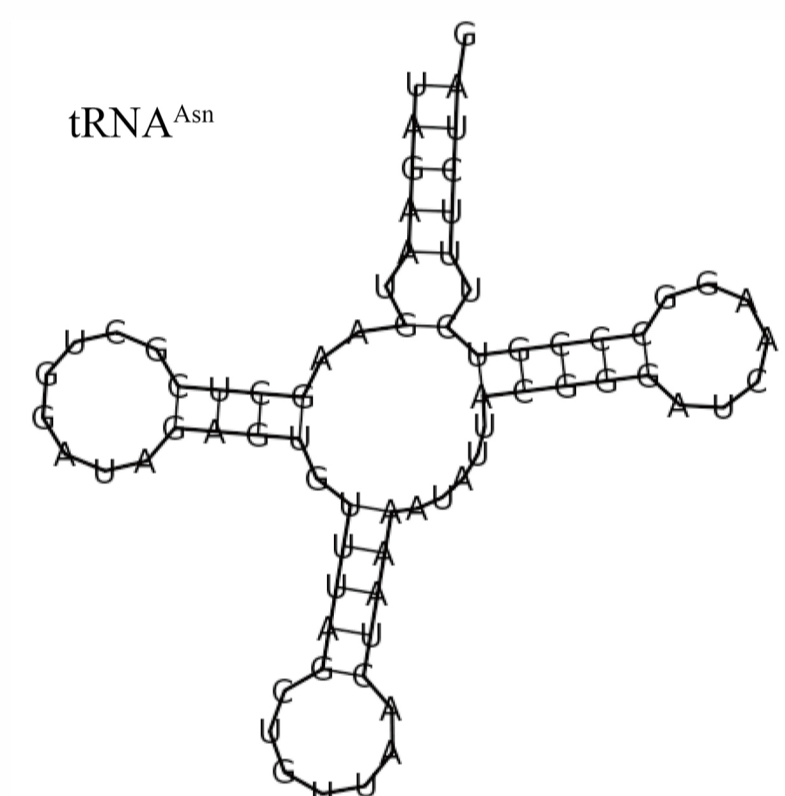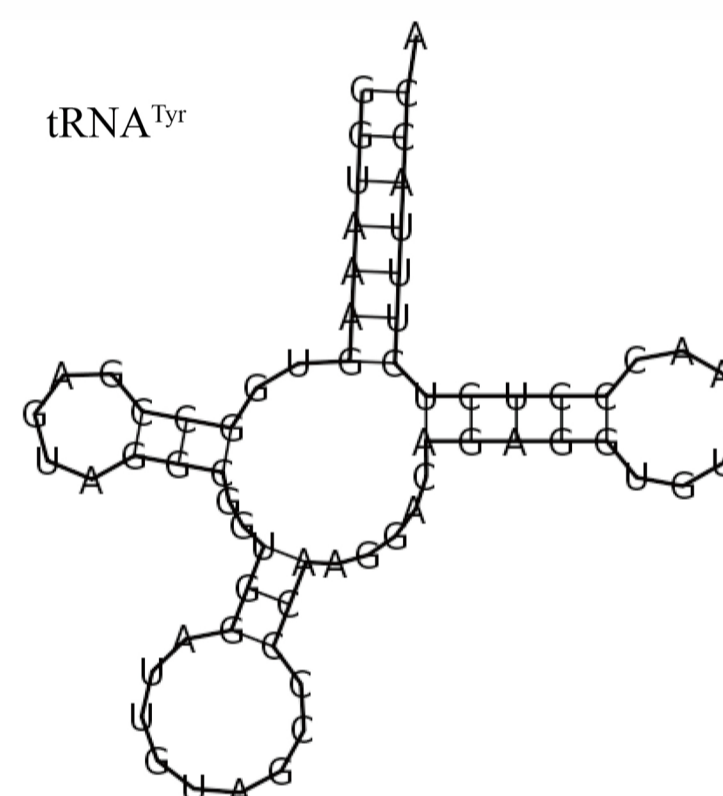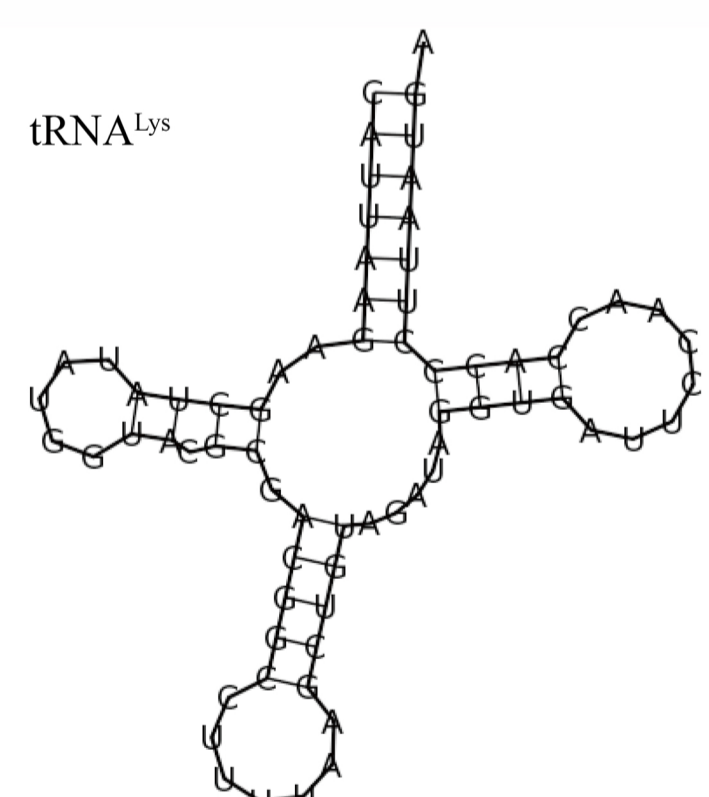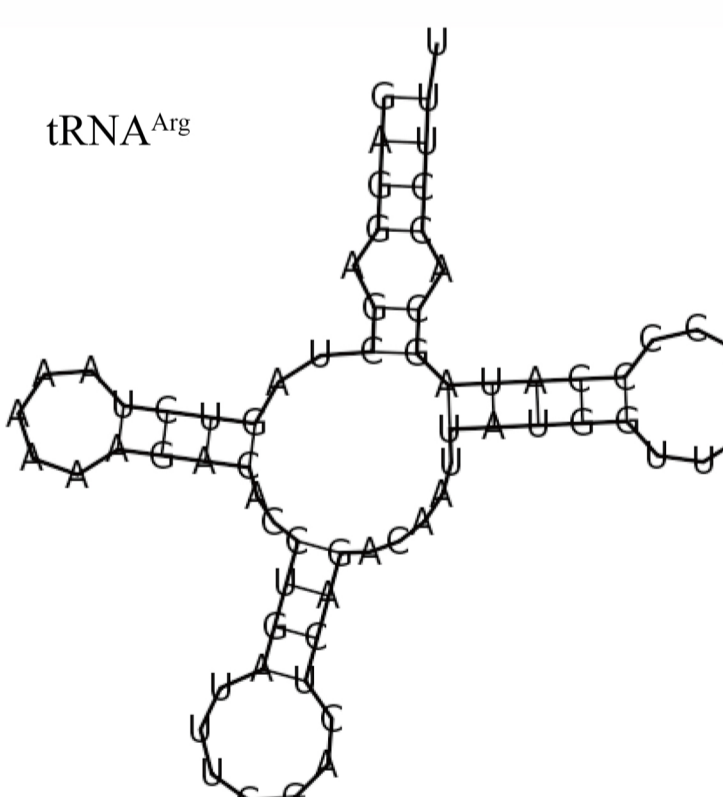

tRNA<sup>His</sup>

Supplement: Figure S1 [file peerj-07-7415-s004.pdf]

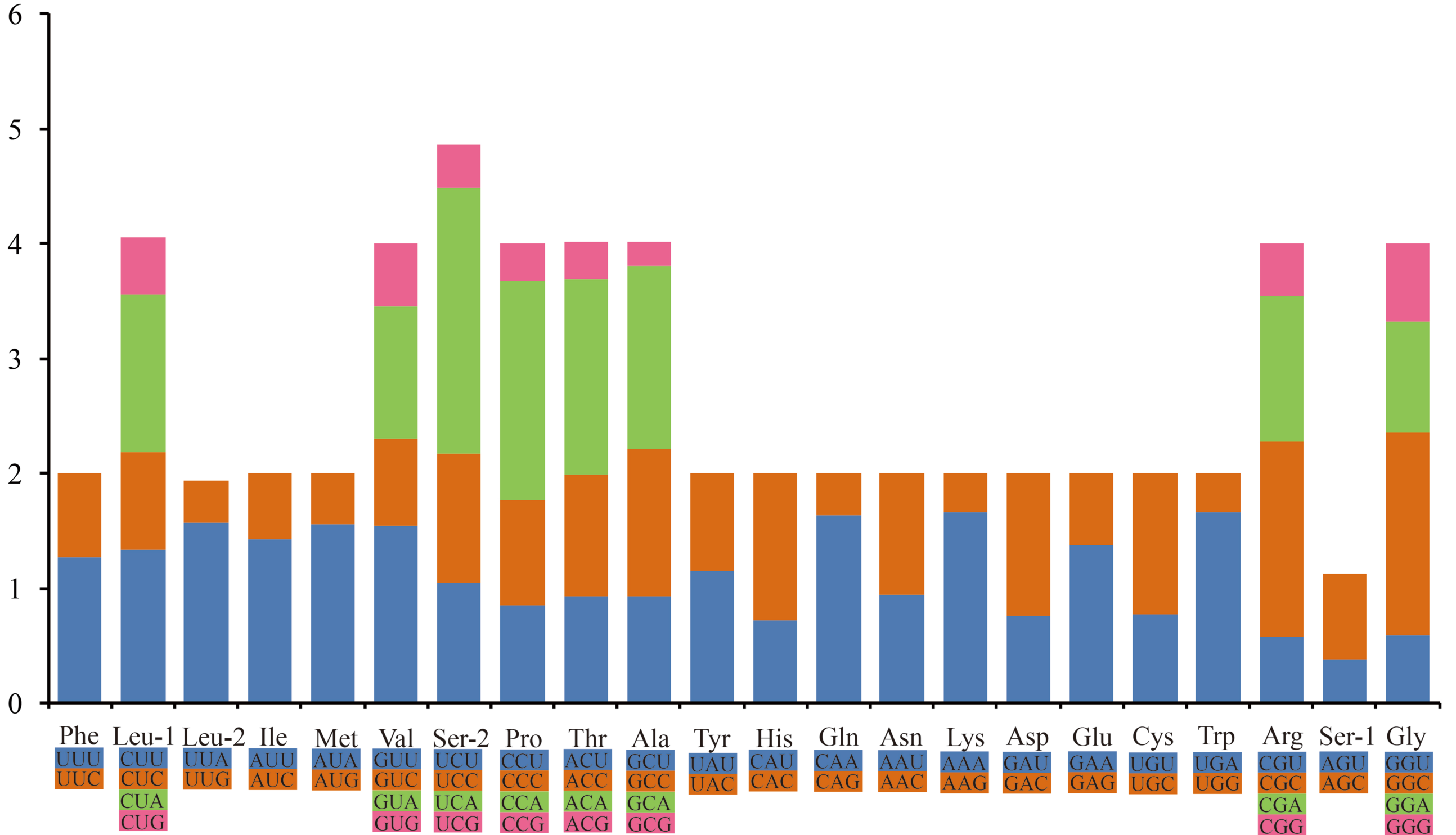

Supplement: Figure S2 — Codon families are provided on the x-axis while RSCU are shown on the y-axis. [file peerj-07-7415-s005.pdf]

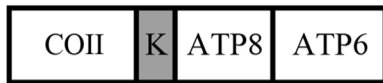

**Tandem duplication**

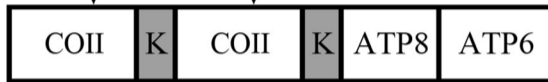

**Random mutation**

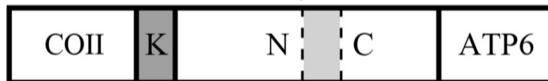

*P. megacephalus*

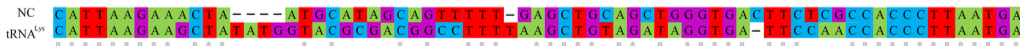

Supplement: Figure S4 — Sequence alignment results shows the noncoding region have high similarity with tRNALys which probably became a pseudogene. [file peerj-07-7415-s007.pdf]
