# Supplementary material for: The revised complete mitogenome sequence of the tree frog Polypedatesmegacephalus (Anura, Rhacophoridae) by next-generation sequencing and phylogenetic analysis
Source: PeerJ. 2019 Aug 1;7:e7415. doi: 10.7717/peerj.7415 (PMC6679912; doi:10.7717/peerj.7415)
Supplement: Figure S3 — The thick solid lines represent replicated genes, dashed lines denote deleted genes, respectively. [file peerj-07-7415-s006.pdf]

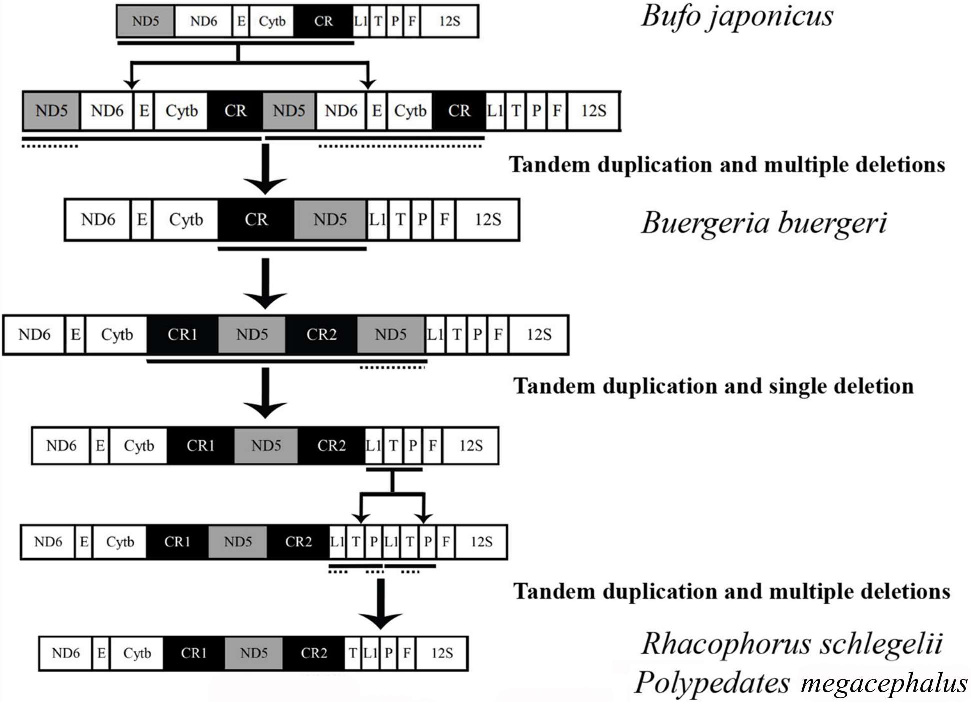

ND5
ND6
E
Cytb
CR
L1
T
P
F
12S
Bufo japonicus
ND5
ND6
E
Cytb
CR
ND5
ND6
E
Cytb
CR
L1
T
P
F
12S
Tandem duplication and multiple deletions
ND6
E
Cytb
CR
ND5
L1
T
P
F
12S
Buergeria buergeri
ND6
E
Cytb
CR1
ND5
CR2
ND5
L1
T
P
F
12S
Tandem duplication and single deletion
ND6
E
Cytb
CR1
ND5
CR2
L1
T
P
F
12S
ND6
E
Cytb
CR1
ND5
CR2
L1
T
P
L1
T
P
F
12S
Tandem duplication and multiple deletions
ND6
E
Cytb
CR1
ND5
CR2
T
L1
P
F
12S
Rhacophorus schlegelii
Polypedates megacephalus
