# Supplementary material for: The revised complete mitogenome sequence of the tree frog Polypedatesmegacephalus (Anura, Rhacophoridae) by next-generation sequencing and phylogenetic analysis
Source: PeerJ. 2019 Aug 1;7:e7415. doi: 10.7717/peerj.7415 (PMC6679912; doi:10.7717/peerj.7415)
Supplement: Figure S5 — Previous sequence was marked by an asterisk (*). Dots represent corresponding nucleotides are identical while dashes indicate gaps in the sequence. Several components of CR are boxed. The downstream of CR* has high similarity with downstream of CR2. [file peerj-07-7415-s008.pdf]

Reapeat I

CR* TAAGCATTAATTTATTTACCCCATCATACTATGTATAATAAGCATTAATTTATTTACCCCATCATACTATGTATAATAAGCATTAATTTATTTACCCCATCATACTATGTATAATAAGCATTAATTTATTTACCCCAT

CR1 ....

CR2 ....

CATACTATGTATAATAAGCATTAATTTATTTACCCCATCATACTATGTATAATAAGCATTAATTTATTTACCCCATCATACTATGTATAATAAGCATTAATTTATTTAAAGTACAATATATAATTTTTTAAATGAACT

....CC.....T.....G.....

....CC.....T.....G.....

TAS

CR* TCAAAGTTTAACATAAAACTATTTAGACTTATAAATGAATGATTTAGAACTAAAATAAAAGAATACATTATTTTATTCAATACATGCTTATTATTTCCATTAAGGGCGGATTCTTAATGAACAACTGATTAATACCAC

CR1 ...G.AA.....ACA.....TC..T..CT...G.....A.....T..C.....C...A.....A.....

CR2 ...G.AA.....ACA.....TC..T..CT...G.....A.....T..C.....C...A.....A.....

TAAACTGAATAAATGATTAAAAATGATTGCCAGAGCCTTAACTATGTCTACTTTATCGGACCTTCACTTGCTTAAGTATATGCGTATCATATCCAAATTTTAATCCACCTCTTTATTCCCATCCCACAAGAATAAATT

.....CCC.....C.....A.....C.A..G.....C.....

.....CCC.....C.....A.....C.A..G.....C.....

CR* AAAATGTATGAATAACAAATAATTATTACTACTTTACACCTTCAAGCATAACACACCTGTTTTAACACAATACTGAATGTATCATTCATTTAAACAAGGTAAGACCTTCACTTGCATTAGATCATGAATATTCCAGTC

CR1 .....T....GG.CT...T..T.....C.....C.....T.....AT.....G.....

CR2 ...G.....T....GG.CT...T..T.....C.....C.....T.....AT.....A.....G.....

AATTTAATATGATAATTAACTATAATGGAATATTATTGACAACAATAACTAATTGCGTTTAAGAAAAAGAGGGCTCTTTTTCTTCAGAATAATTATTCATCTCCTATTATAATAATCCATGAATATTATCTACCAATT

.....A.....C.A.T.....C.....A.....T.....

.....A.....C.A.T.....C.....A.....T.....

CR* GTCTGCATATTCGGACATAACGTCCATCCACTAAACCTAAATTAGCATGCCCTCAACCCAGTAATTATATCATGGACCTCTCGCAGTGCACTCTACCGGGATCAATTAATAAGCCGACCCCTTTCTCTCTTTTCATGA

CR1 A.....A.....C.....A.T.....T..A.....G.-...A.....

CR2 A.....A.....C.....A.T.....T..A.....G.-...A.....

GACTTCTGACGGAACCGAATCTATGGACCCACAGATAAGTATAACCTACTGAAGCTGTTTTAAAGGCTTTGGTGGGCTGGTGAGATAAGGACCTTTAATATAAGCTCAGACCACTTAATGAAGTCAAGGTCGGCCCAG

.G.....T.....TTA....A.C..C.....A.CA.-.....A.....G.....G.....

.G.....T.....TTA....A.C..C.....A.CA.-.....A.....G.....G.....

O_H

CR* ACTATTTTGGGTTTTTGGCATTCATCAGCAAGGTCAGGGTGGTTTCACTCTGAGCGTCATCGCCAATATTATACTACAAGGCACCCACTCAGCAGTGTTCACCTACCCTTCCCATGGACAACAAAAAGCAGGCATTTT

CR1 .....TC.....A

CR2 .....TC.....A

CSB 1

AATTAAGGTGGGGCAAAAAGCTATAATTGATGTCCCGCTAATCTATGGTATCCCCAGGACATCTCTGCATGCTTGTAAGACATATTTTTAACGATCTAGCGAAATCCCCTATACCCTTATTTCCCCCCCCTTTGCACT

.....G.....A....G.....C.....C..G...AC.....

.....G.....A....G.....C.....C..G...AC.....

CSB 2 **CSB 3**

CR* TTTTTTAAACTTTTTTCATTAAAATTTTACAAAATTTAAAAATTTTTCGCGCTACCCCCCCCTTACCCCCCCCCATGGATTATTCTACCAGCAACTCCTAATACCCCCCCCCGGGATTAAGAACTGTTAGATTTTCTGGA

CR1 .....C.....C.....G.....C.....

CR2 .....C.....C.....G.....C.....

Reapeat II

AGGTCCACAGAGCAATGCTGCTTCTGAGTGTATACCTGAATT----CCCCGTAAATATATCATTATGCAATTTCAGTAGATATGTAAATTAATCTTATGAAATTGATCAATTTATATTGATGAATTTAACTAATCTCC

.....A.....TTCC..ATCAC-TA----------

.....A.....TTTT....CCC.TAT....T.....A.....G.C.C.....A.....G.....C.....

CR* GTGATTTCCTGCGTAAATATATCATTATGCAATTTCAGTAGATATGTAAATTAATCTTATGAAATTGATCAATTTATATTGATGAATTTAACTAATCTCCGTGATTTCCTGCGTAAATATATCATTATGCAATTTCAG

CR1 ----------

CR2 T.....T.....C.C.....A.....G.....C.....T.....T.....

TAGATATGTAAATTAATCTTATGAAATTGATCAATTTATATTGATGAATTTAACTAATCTCCGTGATTTCCTGCGTAAATATATCATTATGCAATTTCAGTAGATATGTAAATTAATCTTATGAAATTGATCAATTTA

.....C.C...C.....A.....G.....C.....T.....T.....A.....G.C.C.....A.....

CR* TATTGATGAATTTAACTAATCTCCGTGATTTCCTGCGTAAATATATCATTATGCAATTTCAGTAGATATGTAAATTAATCTTATGAAATTGATCAATTTATATTGATGAATTTAACTAATCTCCGTGATTTCCTGCGT

CR1 ----------

CR2 .....G.....C.....T.....T.....C.C.....A.....G.....C.....T.....

AAATATATCATTATGCAATTTCAGTAGACATGTAAATTAATCTTATGAAATTGATCAATTTAT

.....T.....A....T.....G.C.C.....A.....
