# Supplementary material for: The revised complete mitogenome sequence of the tree frog Polypedatesmegacephalus (Anura, Rhacophoridae) by next-generation sequencing and phylogenetic analysis
Source: PeerJ. 2019 Aug 1;7:e7415. doi: 10.7717/peerj.7415 (PMC6679912; doi:10.7717/peerj.7415)
Supplement: Table S1 [file peerj-07-7415-s009.docx]

**Table S1** **Mitochondrial genome information of Anura species from GenBank**

| Species | GenBank accession no. | Family | Length(bp) | Reference |
| --- | --- | --- | --- | --- |
| *Babina holsti* | NC_022870 | Ranidae | 19113 | *Kakehashi et al., 2013* |
| *Babina subaspera*  *Bombina bombina*  *Bombina maxima* | NC_022871  EU115993  EU789363 | Ranidae  Bombinatoridae  Bombinatoridae | 18525  17154  18388 | *Kakehashi et al., 2013*  *Pabijan et al., 2008*  Unpublished |
| *Buergeria buergeri* | AB127977 | Rhacophoridae | 19959 | *Sano et al., 2004* |
| *Euphlyctis hexadactylus* | NC_014584 | Dicroglossidae | 20280 | *Alam et al., 2010* |
| *Fejervarya cancrivora* | EU652694 | Dicroglossidae | 17843 | *Ren et al., 2009* |
| *Fejervarya limnocharis* | AY158705 | Dicroglossidae | 17717 | *Liu et al., 2005* |
| *Fejervarya multistriata*  *Hyla chinensis*  *Hyla japonica* | KR071859  AY458593  AB303949 | Dicroglossidae  Hylidae  Hylidae | 17750  18180  19519 | *Huang & Tu, 2016*  *Zhang et al., 2005a*  *Igawa et al., 2008* |
| *Hoplobatrachus rugulosus* | NC_019615 | Dicroglossidae | 20309 | *Yu et al., 2012* |
| *Hoplobatrachus tigerinus* | NC_014581 | Dicroglossidae | 20462 | *Alam et al., 2010* |
| *Mantella madagascariensis* | AB212225 | Mantellinae | 22874 | *Kurabayashi et al., 2008* |
| *Occidozyga martensii* | GU177877 | Dicroglossidae | 18321 | *Li et al., 2014a* |
| *Polypedates megacephalus* | MH936677 | Rhacophoridae | 19952 | This study |
| *Rana amurensis*  *Rana kunyuesis* | MF370348  NC_024548 | Ranidae  Ranidae | 20571  22255 | Unpublished  *Li et al., 2014b* |
| *Rhacophorus dennysi* | KT191129 | Rhacophoridae | 17572 | *Huang et al., 2016* |
| *Rhacophorus schlegelii* | AB202078 | Rhacophoridae | 21359 | *Sano et al., 2005* |
