# Supplementary material for: The revised complete mitogenome sequence of the tree frog Polypedatesmegacephalus (Anura, Rhacophoridae) by next-generation sequencing and phylogenetic analysis
Source: PeerJ. 2019 Aug 1;7:e7415. doi: 10.7717/peerj.7415 (PMC6679912; doi:10.7717/peerj.7415)
Supplement: Table S2 [file peerj-07-7415-s010.docx]

**Table S2 The best models of partitioning schemes selected by PartitionFinder for BI analysis.**

| Partitions | Best Model | Sites included in partition |
| --- | --- | --- |
| 1 | GTR+I+G | ND1_pos1 |
| 2 | GTR+I+G | ND3_pos2, ND1_pos2 |
| 3 | GTR+I+G | ND1_pos3 |
| 4 | GTR+I+G | ND2_pos1 |
| 5 | GTR+I+G | ND2_pos2 |
| 6 | GTR+G | ND2_pos3 |
| 7 | GTR+I+G | COI_pos1 |
| 8 | GTR+G | COI_pos2 |
| 9 | GTR+I+G | COI_pos3 |
| 10 | GTR+G | COIII_pos1, COII_pos1 |
| 11 | GTR+G | COII_pos2 |
| 12 | GTR+I+G | COIII_pos3, COII_pos3, ND3_pos3 |
| 13 | GTR+I+G | ATP6_pos1 |
| 14 | GTR+G | ATP6_pos2 |
| 15 | GTR+I+G | ND4_pos3, ATP6_pos3 |
| 16 | GTR+I+G | Cytb_pos2, COIII_pos2 |
| 17 | GTR+I+G | Cytb_pos1, ND3_pos1 |
| 18 | GTR+G | ND4_pos2, ND4L_pos1 |
| 19 | GTR+I+G | ND4L_pos2, ND6_pos2 |
| 20 | GTR+I+G | ND4L_pos3, ND6_pos1, ND5_pos3 |
| 21 | GTR+I+G | ND4_pos1 |
| 22 | GTR+I+G | ND6_pos3 |
| 23 | GTR+I+G | Cytb_pos3 |
| 24 | GTR+I+G | ND5_pos1 |
| 25 | GTR+I+G | ND5_pos2 |
| 26 | GTR+I+G | 12S |
| 27 | GTR+I+G | 16S |

**Note.**

Codon positions are represented by pos1, pos2 and pos3, respectively.
