# Supplementary material for: The revised complete mitogenome sequence of the tree frog Polypedatesmegacephalus (Anura, Rhacophoridae) by next-generation sequencing and phylogenetic analysis
Source: PeerJ. 2019 Aug 1;7:e7415. doi: 10.7717/peerj.7415 (PMC6679912; doi:10.7717/peerj.7415)
Supplement: Table S3 [file peerj-07-7415-s011.docx]

**Table S3 Location of features in the mtDNA of *P.megacephalus***

| **Feature** | **Strand** | **Position** | **Length** | **Initiation** | **Stop** | **Anticodon** | **Intergenic** |
| --- | --- | --- | --- | --- | --- | --- | --- |
|  |  |  | **(bp)** | **codon** | **codon** |  | **nucleotide** |
| tRNA^Thr^ | H | 1-71 | 71 |  |  | TGT |  |
| tRNA^Leu(CUN)^ | H | 72-143 | 72 |  |  | TAG | 5 |
| tRNA^Pro^ | L | 149-217 | 69 |  |  | TGG | 1 |
| tRNA^Phe^ | H | 219-288 | 70 |  |  | GAA | -2 |
| 12S rRNA | H | 287-1216 | 930 |  |  |  |  |
| tRNA^Val^ | H | 1217-1285 | 69 |  |  | TAC |  |
| 16S rRNA | H | 1286-2856 | 1571 |  |  |  | 2 |
| tRNA^Leu(UUR)^ | H | 2859-2932 | 74 |  |  | TAA | 3 |
| ND1 | H | 2936-3896 | 961 | ATG | T(AA) |  |  |
| tRNA^Ile^ | H | 3897-3967 | 71 |  |  | GAT | -1 |
| tRNA^Gln^ | L | 3967-4037 | 71 |  |  | TTG | -1 |
| tRNA^Met^ | H | 4037-4105 | 69 |  |  | CAT |  |
| ND2 | H | 4106-5143 | 1038 | ATT | TAG |  | -2 |
| tRNA^Trp^ | H | 5142-5212 | 71 |  |  | TCA |  |
| tRNA^Ala^ | L | 5213-5282 | 70 |  |  | TGC | 1 |
| tRNA^Asn^ | L | 5284-5356 | 73 |  |  | GTT | 2 |
| OL | L | 5359-5384 | 26 |  |  |  | -1 |
| tRNA^Cys^ | L | 5384-5448 | 65 |  |  | GCA |  |
| tRNA^Tyr^ | L | 5449-5515 | 67 |  |  | GTA | 4 |
| COI | H | 5520-7073 | 1554 | ATA | AGG |  | -13 |
| tRNA^Ser(UCN)^ | L | 7061-7131 | 71 |  |  | TGA | 1 |
| tRNA^Asp^ | H | 7133-7201 | 69 |  |  | GTC |  |
| COII | H | 7202-7891 | 690 | ATA | TAA |  | 5 |
| tRNA^Lys^ | H | 7897-7966 | 70 |  |  | TTT |  |
| NC | H | 7967-8828 | 862 |  |  |  |  |
| ATPase6 | H | 8829-9507 | 679 | ATA | T(AA) |  |  |
| COIII | H | 9508-10291 | 784 | ATG | T(AA) |  |  |
| tRNA^Gly^ | H | 10292-10359 | 68 |  |  | TCC |  |
| ND3 | H | 10360-10699 | 340 | ATG | T(AA) |  |  |
| tRNA^Arg^ | H | 10700-10768 | 69 |  |  | TCG |  |
| ND4L | H | 10769-11053 | 285 | ATG | TAA |  | -7 |
| ND4 | H | 11047-12409 | 1363 | GTG | T(AA) |  |  |
| tRNA^His^ | H | 12410-12478 | 69 |  |  | GTG |  |
| tRNA^Ser(AGY)^ | H | 12479-12546 | 68 |  |  | GCT | 2 |
| ND6 | L | 12549-13040 | 492 | ATG | AGG |  |  |
| tRNA^Glu^ | L | 13041-13109 | 69 |  |  | TTC | 4 |
| Cytb | H | 13114-14269 | 1156 | ATG | T(AA) |  |  |
| CR1 | H | 14270-15843 | 1574 |  |  |  |  |
| ND5 | H | 15844-17622 | 1779 | ATG | TAA |  |  |
| CR2 | H | 17623-19952 | 2330 |  |  |  |  |

**Notes.**

“H”means gene encoded by the H-strand, “L” means gene encoded by the L-strand. Intergenic nucleotide represent noncoding base between genes, a negative number(-) denotes a gene overlapping.
