# Supplementary material for: The revised complete mitogenome sequence of the tree frog Polypedatesmegacephalus (Anura, Rhacophoridae) by next-generation sequencing and phylogenetic analysis
Source: PeerJ. 2019 Aug 1;7:e7415. doi: 10.7717/peerj.7415 (PMC6679912; doi:10.7717/peerj.7415)
Supplement: Table S4 [file peerj-07-7415-s012.docx]

**Table S4 Base compositions of the mitochondrial genome of** ***Polypedates*** ***megacephalus***

| **Region** | **A%** | **C%** | **G%** | **T%** | **A+T%** | **G+C%** | **AT skew** | **GC skew** |
| --- | --- | --- | --- | --- | --- | --- | --- | --- |
| Whole genome | 30.2 | 24.6 | 14.8 | 30.4 | 60.6 | 39.4 | -0.003 | -0.249 |
| 12S rRNA | 32.9 | 22.9 | 19.4 | 24.8 | 57.7 | 42.3 | 0.140 | -0.083 |
| 16S rRNA | 34.6 | 22.2 | 17.7 | 25.6 | 60.2 | 39.9 | 0.150 | -0.113 |
| ND1 | 28.7 | 26.0 | 13.0 | 32.3 | 61.0 | 39.0 | -0.059 | -0.333 |
| ND2 | 31.7 | 25.7 | 11.2 | 31.4 | 63.1 | 36.9 | 0.005 | -0.394 |
| COI | 23.4 | 25.2 | 18.6 | 32.8 | 56.2 | 43.8 | -0.168 | -0.150 |
| COII | 30.1 | 24.1 | 16.5 | 29.3 | 59.4 | 40.6 | 0.013 | -0.187 |
| ATP6 | 27.1 | 27.7 | 12.2 | 33.0 | 60.1 | 39.9 | -0.097 | -0.388 |
| COIII | 27.2 | 24.9 | 16.8 | 31.1 | 58.3 | 41.7 | -0.067 | -0.194 |
| ND3 | 22.6 | 27.6 | 17.9 | 31.8 | 54.4 | 45.5 | -0.169 | -0.212 |
| ND4L | 21.8 | 27.0 | 16.8 | 34.4 | 56.1 | 43.9 | -0.225 | -0.232 |
| ND4 | 28.1 | 24.7 | 13.7 | 33.5 | 61.5 | 38.4 | -0.087 | -0.286 |
| ND6 | 39.2 | 33.9 | 10.6 | 16.3 | 55.5 | 44.5 | 0.413 | -0.524 |
| Cytb | 27.8 | 26.7 | 14.0 | 31.5 | 59.2 | 40.7 | -0.062 | -0.312 |
| CR1 | 33.3 | 22.2 | 12.1 | 32.4 | 65.7 | 34.3 | 0.014 | -0.294 |
| ND5 | 30.2 | 28.5 | 12.7 | 28.6 | 58.8 | 41.2 | 0.029 | -0.383 |
| CR2 | 33.2 | 19.6 | 11.7 | 35.5 | 68.8 | 31.3 | -0.033 | -0.252 |
